# Targeting intracellular B2 receptors using novel cell-penetrating antagonists to arrest growth and induce apoptosis in human triple-negative breast cancer

**DOI:** 10.18632/oncotarget.24009

**Published:** 2018-01-05

**Authors:** Céléna Dubuc, Martin Savard, Veronica Bovenzi, Andrée Lessard, Audrey Fortier, Jérôme Côté, Witold Neugebauer, Flavio Rizzolio, Sameh Geha, Antonio Giordano, Sylvain Chemtob, Fernand Gobeil

**Affiliations:** ^1^Department of Pharmacology and Physiology, Faculty of Medicine and Health Sciences, Université de Sherbrooke, Sherbrooke, Québec, Canada; ^2^Institute of Pharmacology, Université de Sherbrooke, Sherbrooke, Québec, Canada; ^3^Department of Psychiatry, Maryland Psychiatric Research Center, University of Maryland School of Medicine, Baltimore, MD, United States; ^4^Department of Biology, Sbarro Institute for Cancer Research and Molecular Medicine, Temple University, Philadelphia, PA, USA; ^5^Dipartimento di Scienze Molecolari e Nanosistemi, Università Ca' Foscari Venezia, Mestre-Venezia, Italy; ^6^Department of Pathology, Centre Hospitalier Universitaire de Sherbrooke, Sherbrooke, Québec, Canada; ^7^ Department of Pediatrics, Centre Hospitalier Universitaire Sainte-Justine Research Center, Montréal, Québec, Canada

**Keywords:** triple-negative breast cancer, kinins, cell-permeable antagonist, nuclear GPCRs, intracrine signaling

## Abstract

G protein-coupled receptors (GPCRs) are integral cell-surface proteins having a central role in tumor growth and metastasis. However, several GPCRs retain an atypical intracellular/nuclear location in various types of cancer. The pathological significance of this is currently unknown. Here we extend this observation by showing that the bradykinin B2R (BK-B2R) is nuclearly expressed in the human triple-negative breast cancer (TNBC) cell line MDA-MB-231 and in human clinical specimens of TNBC. We posited that these “nuclearized” receptors could be involved in oncogenic signaling linked to aberrant growth and survival maintenance of TNBC. We used cell-penetrating BK-B2R antagonists, including FR173657 and novel transducible, cell-permeable forms of the peptide B2R antagonist HOE 140 (NG68, NG134) to demonstrate their superior efficacy over impermeable ones (HOE 140), in blocking proliferation and promoting apoptosis of MDA-MB-231 cells. Some showed an even greater antineoplastic activity over conventional chemotherapeutic drugs *in vitro*. The cell-permeable B2R antagonists had less to no anticancer effects on B2R shRNA-knockdown or non-B2R expressing (COS-1) cells, indicating specificity in their action. Possible mechanisms of their anticancer effects may involve activation of p38kinase/p27^Kip1^ pathways. Together, our data support the existence of a possible intracrine signaling pathway via internal/nuclear B2R, critical for the growth of TNBC cells, and identify new chemical entities that enable to target the corresponding intracellular GPCRs.

## INTRODUCTION

Breast cancer (BC) is the most frequently diagnosed cancer in North America and figures among the leading causes of cancer deaths in women worldwide [1]. This situation remains true despite improvements in screening and treatment. Non-invasive forms of BC most often originate from the galactophorous ducts and lobules (the milk-producing glands) and are classified as ductal carcinoma *in situ* (DCIS) or lobular carcinoma *in situ* (LCIS). They are generally benign tumors accounting for approximately 20% and 1% of all newly diagnosed BC cases, respectively [2]. These tumors are highly curable if not very manageable cancers but they are seen as precursors of invasive BC. Invasive ductal carcinoma is the most common type of BC, representing 65 to 85% of all cases. Current treatment options and prognosis for invasive BC vary depending on various factors, including the histopathological type, grade, stage and steroid receptors (estrogen and progesterone receptors (ER and PR)) and epidermal growth factor receptor-2 (HER2/neu) status. Currently available treatments include some combination of surgery, radiation, chemotherapy, hormonal therapy, and targeted therapies including the use of monoclonal antibodies (e.g. trastuzumab; Herceptin^®^) notably for HER-2-positive patients [3].

The triple-negative breast cancer (TNBC), defined by the absence of ER, PR and HER2 expression, accounts for 10–20% of newly diagnosed cases of invasive BC. TNBC encompasses a remarkably genetically heterogenous group of tumors with different clinicopathological features. It is associated with aggressive growth and increased risk of local recurrence and distant metastasis (brain and lung), and of developing high resistance to chemotherapy. For these reasons, it remains the hardest BC subtype to treat and prognosis is poor compared to all BC subtypes [4]. In fact, the overall 5-year rate for patients with late-stage (metastatic) TNBC is less than 30%, despite chemotherapy, the mainstay of adjuvant treatment for this condition [3, 4]**.** The lack of effective treatments for TNBC warrants the identification of new molecular targets and approaches to develop efficient therapeutic agents for the treatment of TNBC.

G protein-coupled receptors (GPCRs) are integral cell-surface proteins having a central role in tumor growth, invasion and metastasis, angiogenesis and chemotherapy resistance [5, 6]. Among those implicated in BC progression, in particular, include thrombin-PAR-1, PGE_2_-EP2/EP4, SDF1-CXCR4, oestrogen-GPR30 and kinin B1/B2 receptors (B1R/B2R) [5, 7, 8]. Given their major contribution to tumor development and progression, they represent promising therapeutic targets for developing next-generation anticancer therapies [6]. However, many GPCRs retain an atypical intracellular/nuclear location in various types of cancer, distinct from its classical location on plasma membrane [9, 10]. The pathological significance of this is currently unknown. Moreover, immunological profiling studies, performed on clinical biopsy specimens from cancer patients, suggested that the nuclear presence of some GPCRs (*e.g.*, CXCR4 [11], CysLT1 [12], EMR-2 [13], EP1 receptor [14]), in analogy to nuclear receptor tyrosine kinases (*e.g.*, EGFR [15, 16] and VEGFR1 [17]), might carry predictive and prognostic values in malignancies as in the case of breast, uterine and prostate cancers. Atypical pattern of increased intracellular/nuclear subdistribution encountered in some types of cancer may be functionally significant in the process of tumor growth. Indeed, there is compelling evidence suggesting that GPCRs can also function intracellularly on endoplasmic reticulum/nuclear membranes to promote noncanonical signaling in health and diseases such as cancer [9, 10, 18–20]. Such signaling mechanism of GPCRs, acting within intracellular space at subcellular organelles, is known as intracrine signaling [19]. Intracellular/nuclear GPCRs may be 1) constitutively active, 2) activated by internal, newly synthesized ligands avoiding secretory pathways, or 3) activated by internalized extracellular ligands through mechanisms dependent or not of receptor-mediated endocytosis and endosomal escape of ligands [10, 19, 21–23].

**Figure 1:**
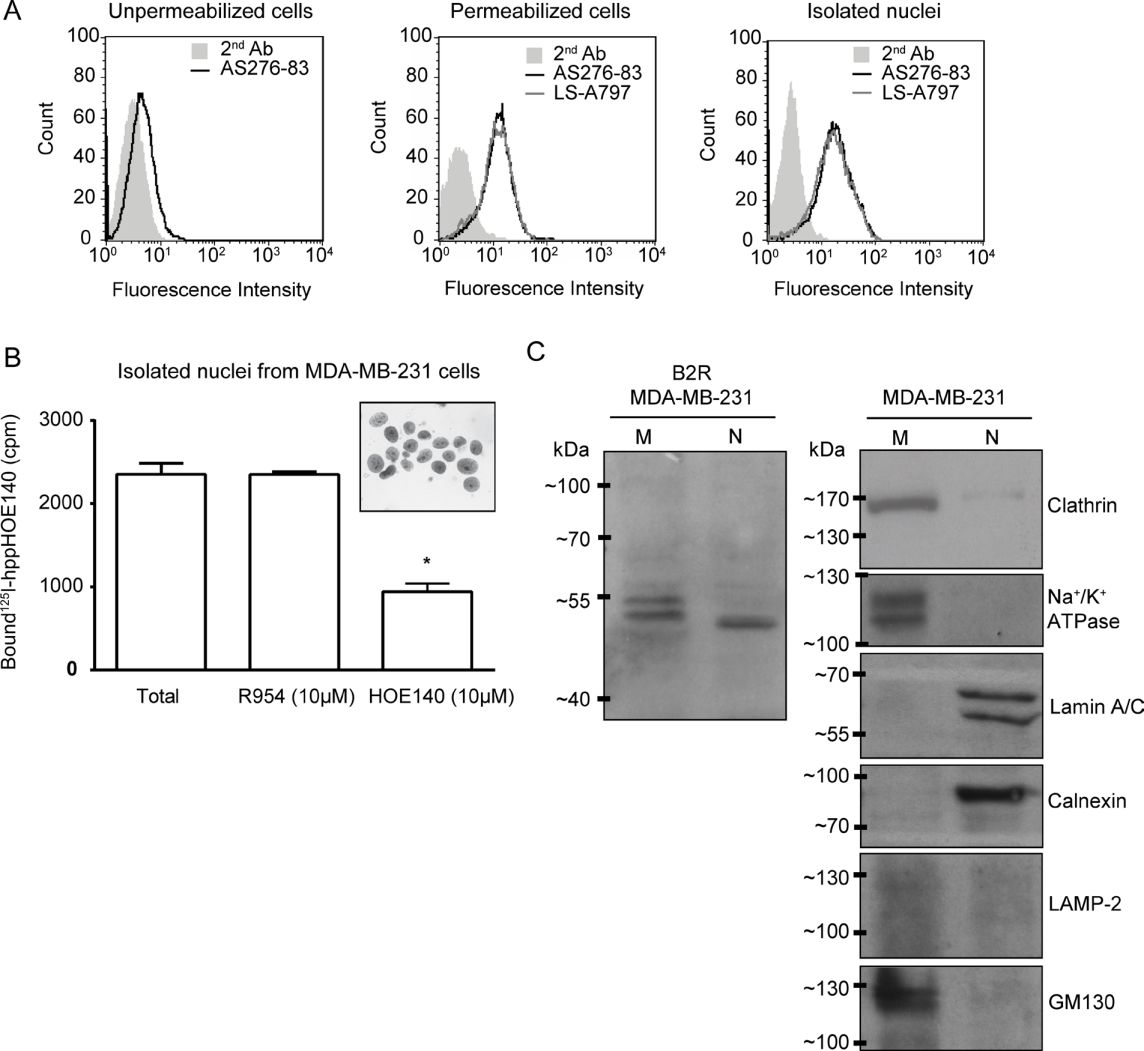
Cellular/nuclear expression of B2R in the human TNBC cell line MDA-MB-231 (**A**) Immunostaining of B2R on unpermeabilized and permeabilized (saponin-treated) MDA-MB-231 cells and on isolated nuclei derived from these cells analyzed by FACS. Experiments were performed using two distinct anti-B2R antibodies: AS276–83 and LS-A797. The second antibody was Alexa Fluor 488^®^ dye conjugated goat anti-rabbit antibody. One out of two representative experiments is shown. (**B**) Competitive binding of radiolabeled HOE140 (0.5 nM) with R954 (10 µM) and HOE140 (10 µM) on nuclei isolated from MDA-MB-231 cells. ^*^p < 0.05 versus indicated group (unpaired Student’s *t* test). Inset. Micrograph depicting the purity ( > 90%) of the nuclear fractions. (**C**) Detection of B2R in purified fractions of plasma membrane (M) and nuclei (N) by western blotting. A repetition of the experiment gave similar results. Prepared nuclear fractions demonstrated lack of immunoreactivity with the organelle specific antibodies to Clathrin and Na^+^/K^+^ATPase (plasma membrane), LAMP-2 (lysosome) and GM130 (cis-Golgi), and high immunoreactivity with lamin A/C (nucleus) and calnexin (endoplasmic reticulum). Conversely, membrane microsomes exhibited high immunoreactivity against Clathrin, Na^+^/K^+^ATPase and GM130, and no immunoreactivity to lamin A/C, calnexin and LAMP-2.

Kinins are a family of peptides which exert regulatory control over cellular processes important for tumor growth, survival, invasion, and angiogenesis of several kinds of cancers [7, 24]. In particular, BK, acting through the GPCR B2R, is implicated in neoplastic growth. In accordance, antagonism of B2R was shown to impair tumor cell proliferation, migration and invasiveness in some preclinical models of cancer [7, 24, 25]. However, these findings could apply to some but not all of the various B2R antagonists studied (*e.g.,* active BKM570, B9870 vs non-active B9430) [24, 25]; an observation that could be attributed not only to differences in their pharmacological activities but also to specific particular physicochemical characteristics between these antagonists, which might affect their movement across cell membrane. Consistent with the notion that kinins may act in an autocrine/intracrine fashion to regulate gene expression is the presence of elevated nuclear B1R and B2R levels as well as the plasma (hKB1) and/or tissue (hK1) kinin forming enzyme kallikreins, that have been reported in certain types of human cancer, such as malignant pleural mesotheliomas [26], lung cancer [27], breast cancer [28] and high-grade [WHO grade IV] gliomas [9]. Results from our exploratory investigation showed that nuclear B2R are prominently expressed in the aggressive, TNBC cell line MDA-MB-231 and in TNBC clinical specimens (Figure 1). Collectively, these findings have led us to believe that nuclearly-located B2R may have an important role to play in the overall functions of the receptors contributing to the growth of TNBC; accordingly, cell-penetrating selective B2R antagonists (CP-B2RAs) are critical to validate function and provide the required tools in developing drug prototypes. In the present study, we used novel cell-permeable (transducible) forms of peptide as well as non-peptidic B2R antagonists as pharmacological tools to explore internal/nuclear B2R activity in MDA-MB-231 cells as a representative *in vitro* TNBC model [29]. Our findings show that a multi-compartment targeting approach (i.e. to plasma and nuclear membranes) by means of CP-B2RAs can be used to enhance killing of tumor cells.

## RESULTS

### Overexpression and nuclear localization of B2R in the agressive TNBC cell line MDA-MB-231 and in clinical TNBC specimens

We first sought to examine the expression and subcellular localization of B2R in MDA-MB-231 cells by FACS using the specific antisera AS276–83, which recognize both intracellular and extracellular domains of B2R [30]. These experiments were made under non-permeabilized and permeabilized conditions for labeling cell-surface and intracellular B2R, respectively. B2R immunostaining was identified on the plasma membrane but was more abundant in intracellular compartments (Figure 1A). These results agree with those obtained by Morissette *et al.* who showed that MDA-MB-231 cells displayed low cell-surface levels of B2R [31]. Interestingly, we found that nuclei isolated from MDA-MB-231 cells similarly stained intensely (Figure 1A). The presence of specific BK receptor sites in purified nuclei isolated from MDA-MB-231 cells was confirmed by marking the B2R with a distinct anti-B2R primary antibody (LS-A797) (Figure 1A). The specificities of the two primary antibodies used were priorly established elsewhere [30, 32, 33] and further described herein by immunoblotting of transfected cells and by FACS analysis on tissues obtained from wild-type and knock-out mice (Supplementary Figure 1). Moreover, competition-binding studies on isolated MDA-MB-231 nuclei using [^125^I]-hpp-HOE 140 (B2R antagonist) indicated binding sites with a pharmacological profile typical of B2R as the unlabeled B2RA HOE 140, but not the B1RA R-954, was able to displace the bound radioligand (Figure 1B).

To further substantiate the results reporting nuclear localization of B2R, we carried out western blot analysis on enriched cell membranous and nuclear fractions from MDA-MB-231 cells following proper validation of these organelle fractions (Figure 1C). We detected a specific anti-B2R immunoreactive band in the nucleus closely matching those detected in plasma membranes, the latter of which appear as two protein doublets, representing possibly different glycosylated protein isoforms (Figure 1C) [33, 34]. These results demonstrate that the full-length B2R localised to nuclei and are competent to bind cognate ligands.

**Figure 2:**
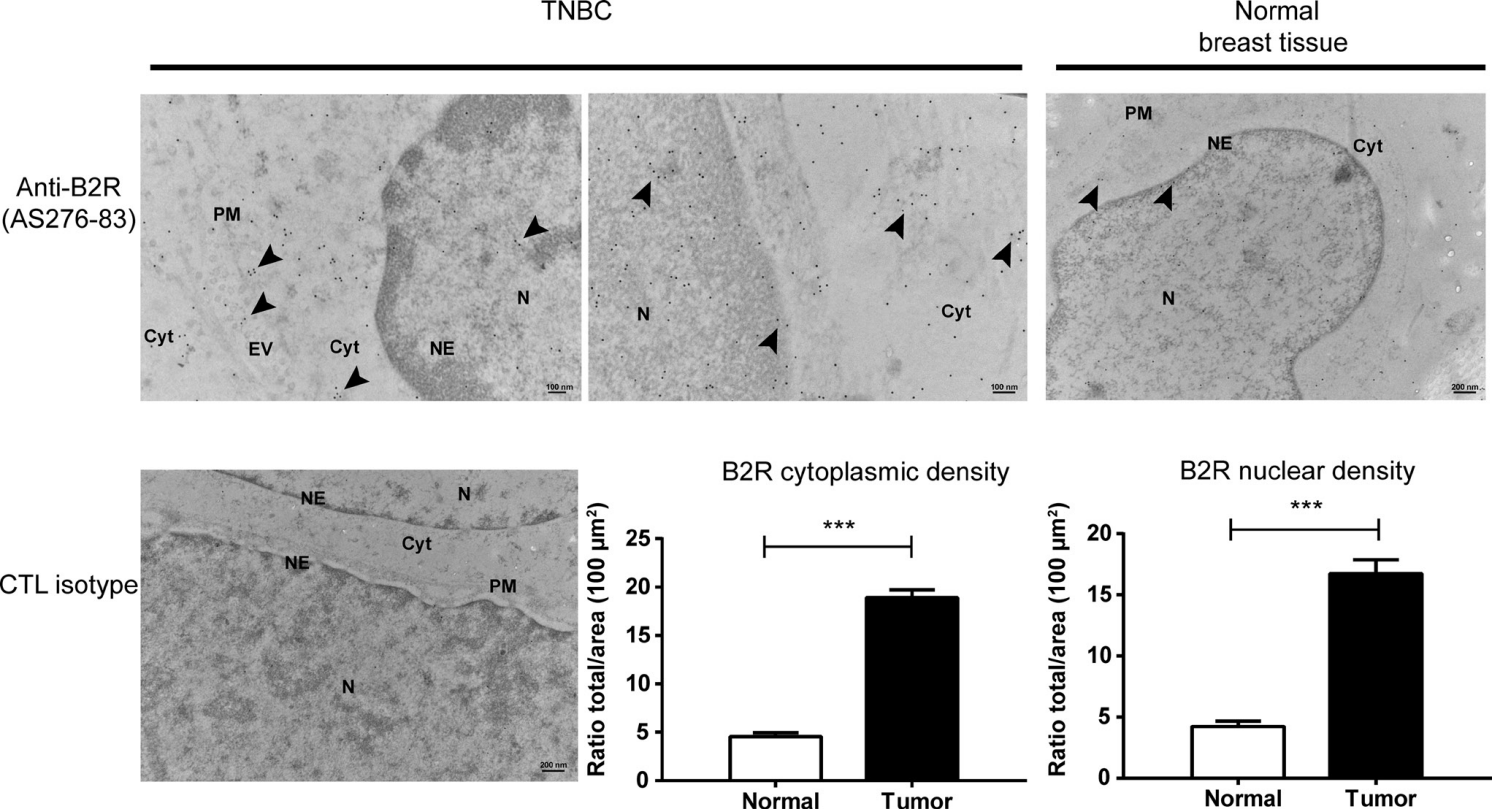
Overexpression and nuclear localization of B2R in clinical TNBC specimens Detection of B2R in TNBC and normal adjacent breast tissue biopsies was assessed using transmission electron microscopy. Positive immunogold B2R labeling at the cell nucleus (N) and nuclear envelopes (NE), cytoplasm (Cyt), plasma membrane (PM) and on extracellular vesicles (EV) (arrow heads). No staining was observed using the isotype control antibody. Representative electron micrographs of human normal breast and TNBC samples stained with a specific antiserum against B2R are shown. Histographic representation of B2R quantification in electron microscopic micrographs showing high receptor density in TNBC, at both cytoplasmic and nuclear compartments, compared to normal tissue samples adjacent to tumors. ^***^p < 0.001 versus normal tissues, as determined with Student’s *t*-tests.

In an initial effort to translate the *in vitro* findings to human, we also examined the expression of B2R in few clinical BC specimens tested negative for ER, PR and HER2, and in adjacent normal tissue samples taken as controls. For this purpose, we used high-resolution immunogold electron microscopy to ascertain the *in situ* subcellular localization of B2R in these human tissues (Figure 2). We found overall higher B2R immunogold labeling in TNBC specimens compared to matched-normal breast tissues. As expected, the presence of B2R was visualized at the cell surface and intracellularly (presumably in vesicles) in TNBC cells (Figure 2). The number of gold particles detected at the plasma membrane components of normal and TNBC tissues were judged too small and inconsistent to find important differences as significant.

Most interestingly, we also detected B2R at extracellular vesicles, also refered to as shedding microvesicles or exosomes, pointing out to expanded roles for B2R in TNBC. It is believed that these tumor-derived exosomes can accelerate cancer progression and metastasis by allowing intercellular transfer of membrane and soluble proteins and microARNs in microenvironmental recipient cells [35].

Quantitatively, marked differences appeared in the nuclear and cytoplasmic distribution of B2R between normal and TNBC tissues (Figure 2); B2R density being about 3-fold higher in TNBC than normal at both compartments. These EM data also confirmed the striking variation in the subcellular patterns of expression of B2R in normal and TNBC. Collectively, these results raise the possibility that an intracellular/nuclear pool of B2R may exist and be biologically relevant for TNBC growth.

### Assessment of pharmacological and cell-penetrating properties of B2RAs

It is recognized that hydrophilic, polar peptide molecules, like BK, do not readily cross cell membrane on their own. Different strategies can however be employed to facilitate their cellular entry into living cells/tissues and ensuing binding to cognate receptors residing inside the cells. Among these, peptide linkage or conjugation with non-immunogenic, non-toxic transduction vectors, consisting of lipids, oligosaccharides and cationic peptides, such as the well-known HIV-1 Tat peptide (48–58 sequence) has been found useful [36–38]. To address putative functions of intracellular B2R, we took into account some of these strategies and designed two dTat-based kinin peptide analogs of BK and HOE 140 (Table 1). To increase the lipophilicity and transcellular permeability of HOE 140, we also designed a novel delivery vector composed of steroidal cholic acid, having no known ability to activate soluble steroid/nuclear receptors and/or GPCRs recognized by lipid mediators [39]. Our data from radioligand binding assays (on adherent HEK-293T cells transfected with B2R) and functional studies (isometric contraction of human vessels) revealed that covalent conjugation of B2RA HOE 140 with the selected carrier vectors did not influence its binding and activity at the cellular level (Table 1). Only dTat-BK showed reductions in both binding affinity (110 ± 4 nM vs 4.9 ± 0.6 nM) and biological activity (350 ± 1 nM vs 3.0 ± 0.4 nM) compared to BK *in vitro*. Direct measument of intracellular contents of the B2RAs by LC-MS/MS were made after different incubation periods with MDA-MB-231 cells to determine their permeability characteristics (Table 1). These assays were complemented by additional measures of their corresponding log D_7.4_ values. Our results showed that the non-peptide B2RA FR173657, as well as the lipopeptide B2RA Cholic-HOE 140 (NG134), can translocate across the plasma membrane into the cell cytoplasm, albeit with quite different uptake rates (Table 1). These are in accordance with the high distribution coefficients measured for these latter antagonists (log D_7.4_ > 0), supporting their passive transport across cell membranes (Table 1). As anticipated, the dTat-HOE 140 conjugate (NG68) also accumulated over time within MDA-MB-231 cells as opposed to its unconjugated parent peptide HOE 140 (Table 1).

**Table 1:** Human kinin B2R binding and pharmacological activities, distribution coefficient and cell permeability values of agents used in the study

Agents / Code name	Binding hB2R-HEK293T IC_50_ (nM)	Bioassay hUV IC_50_ (nM)	Distribution coefficient log D, n-octanol/PBS (pH 7.4)	Cellular incorporation (pmol/10^6^ cells)		
				15 min	4 h	24 h
HOE 140	5.3 ± 1.7^*^	3.8 ± 0.3^*^	-3.06	0	0	0
FR 173657	37.0 ± 1.6^*^	6.0 ± 0.8^*^	2.89	116	139	185
d-Tat-HOE 140 (NG68)	5.2 ± 1.0	18.0 ± 4.0	-3.02	5	14	21
Cholic-HOE 140 (NG134)	19.0 ± 6.5	6.5 ± 2.5	0.21	13	22	63

Values are means ± s.e.m. ^*^Data taken from ref. [34]. Binding assays were performed using living adherent HEK-293T cells transiently transfected with hB2R. Pharmacological activities of B2RAs were performed on human de-endothelized umbilical veins (hUV). Distribution coefficient of compounds between n-octanol and 0.1 M sodium phosphate buffer, pH 7.4, was determined by the shake flask methodology. Compound content of the aqueous and octanol phases was quantified by RP-HPLC. Cellular incorporation of B2RAs within MDA-MB-231 cells were assessed using a tandem quadrupole LC-MS/MS system. Results are the average of two independent assays.

**Figure 3:**
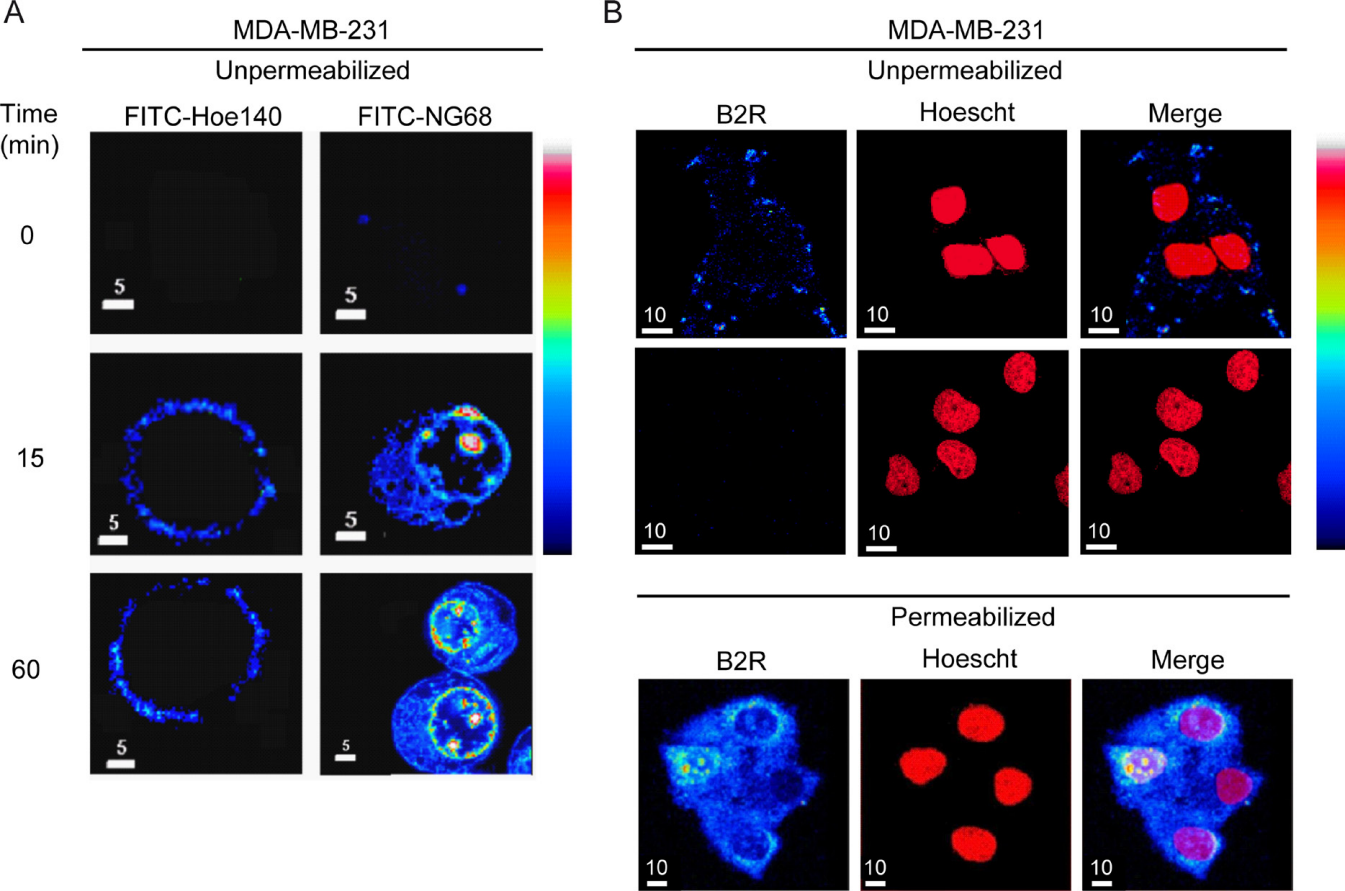
Binding and cellular incorporation of B2RAs in MDA-MB-231 cells (**A**) MDA-MB-231 cells were incubated with FITC-labelled peptide HOE140 or NG68 (10 µM) at 37°C for the indicated times, washed with PBS buffer, fixed with paraformaldehyde then analyzed by laser confocal microscopy. A pseudocolor fluorescence intensity scale is shown on the right; black being the lowest and white the highest value. Note the rapid cellular incorporation of FITC-NG68 peptide and its perinuclear/nucleolar localization increasing over time while FITC-HOE140 conserved an exclusive membrane distribution. No signal was detected when cells were incubated with FITC alone under the same experimental conditions. Individual midsection confocal images are representative of three replicates. (**B**) Immunocytochemical localization of B2R in MDA-MB-231 cells was performed in unpermeabilized (upper panels) and permeabilized (bottom panels) conditions using the B2R antiserum AS276–83. An Alexa Fluor 568-conjugated goat anti-rabbit IgG antibody was used as a secondary antibody. Nuclei stained with Hoescht 33342 (middle panels, arbitrary colors). Merge in right panels. Negative control: no primary antibody. Pseudocolor fluorescence intensity scale as in A. Representative confocal middle z-sections are shown.

Using confocal microscopy, we further demonstrated the ability of fluorescent dTat-HOE 140 conjugate (NG68) to readily gain access to intracellular sites then progressively to nuclear compartments of MDA-MB-231 cancer cells whereas the unconjugated HOE 140 can only bind to cell surface receptors (Figure 3A). These results closely resembled those obtained by immunofluorescence, under nonpermeabilized or permeabilized conditions, showing the small fine-punctate appearance of B2R immunoreactivity along the cell surface of MDA-MB-231 cells, while the strongest staining being observed intracellularly at the perinuclear and nucleolar regions (Figure 3B).

### CP-B2RAs impaired clonogenic / proliferative potential of MDA-MB-231 cells

**Figure 4:**
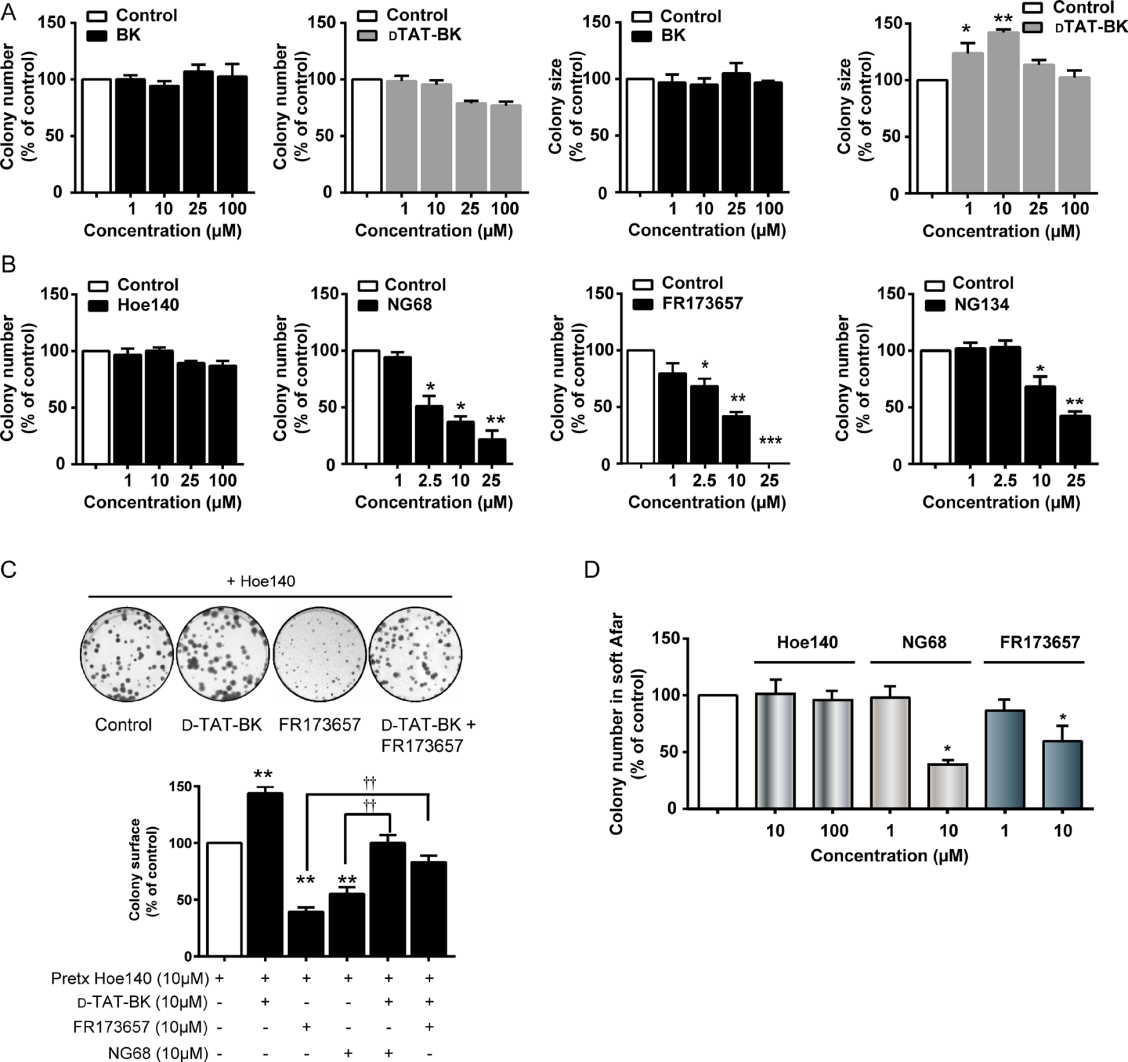
CP-B2RAs abrogate clonogenic potential of MDA-MB-231 cells (**A**) Effects of B2R agonists on colony size and numbers determined by anchorage-dependent clonogenic assays. Cells were incubated in the presence or absence of indicated concentrations of agonists and colonies allowed to form over a 10–14 day period. (**B**) Effects of B2RAs on colony numbers. Experiments performed as described in A. DMSO vehicle control (0.2%), serving the maximal concentration tested of FR173657 (25 µM), did not have any marked effects on clonogenicity (not shown). (**C**) Growth competition assays with CP-B2R agonist dTat-BK and CP-B2RAs, NG68 and FR173657. Cells were first incubated with a saturating concentration of HOE 140 (to selectively block cell-surface B2R) then treated with the dTat-BK, with or without CP-B2RAs, at the indicated concentrations. Representative photographs of stained colonies following the indicated treatments are shown above graph. (**D**) Effects of B2RAs on cell growth assessed by anchorage-independent (in soft agar) clonogenic assays. Cells were exposed to the indicated concentrations of compounds for 7 days. Colony formation in soft agar was assayed as described in Materials and Methods. For panels A-D, data represent means ± s.e.m. of 4–8 experiments. ^*^p < 0.05, ^**^p < 0.01, ^***^p < 0.001 versus Ctl (Dunnett’s test); ^††^*p* < 0.01 versus indicated group (unpaired Student’s *t* test).

With these new tools at hand, we then proceeded in comparing the effects of membrane permeant and non permeant B2R ligands on cell proliferation/growth of TNBC cells using clonogenic assays. Colony number and surface area were both used as clonogenic assay endpoints to evaluate the effects of agonists (Figure 4A). Incubation of MDA-MB-231 cells with the biologically active, transducible B2R agonist dTat-BK did not increase colony number, but resulted in an increase in colony surface area, with a forming bell-shaped concentration-response curve. The causes behind such hormetic effect are unclear. Comparatively, the unmodified parent peptide agonist BK was ineffective. In addition, we found that only the cell permeable B2R antagonists, NG68, NG134 and FR173657 showed concentration-responsive clonal repression. HOE140 (up to 100 μM) did not prevent colony formation (Figure 4B), even when added on a daily basis for a period of 10 days (not shown). Similar results were found using other human B2R-expressing carcinoma cell lines, such as U87-MG glioblastoma and A549 lung cancer cells (Supplementary Figure 2). Furthermore, blocking cell-surface B2R with a saturating concentration of HOE140 (10 μM) prior to treatment with the CP-B2RAs did not affect their inhibitory activity against cell proliferation (Figure 4C).

**Figure 5:**
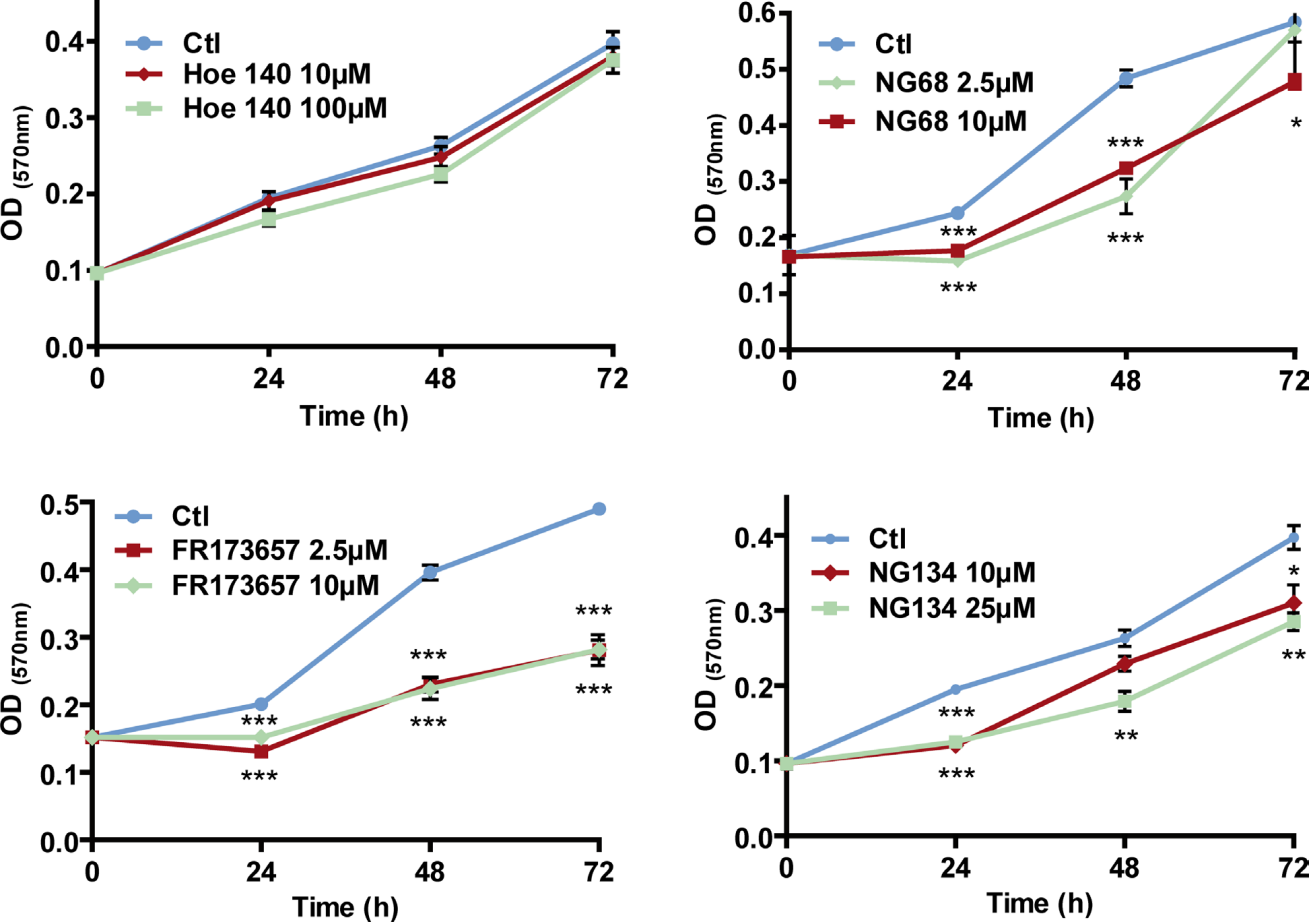
Concentration-dependent anti-proliferative effects of CP-B2RAs on MDA-MB-231 cells Cell proliferation assay was determined with colorimetric MTT as described under Materials and Methods. Data are means ± s.e.m. of optical density values from 5 to 8 experiments. ^*^p < 0.05, ^**^p < 0.01, ^***^p < 0.001 versus control (untreated) group at corresponding time point, using unpaired Student’s *t* test.

In another set of experiments, we studied the possible competition for intracellular receptor binding sites between the B2R agonist and antagonist. For this, we used the non-permeable peptide antagonist HOE 140 (in excess) to selectively block cell-surface B2R in MDA-MD-231 cells while exposing intracellular/nuclear B2R with the membrane pemeant agonist dTat-BK, with or without CP-B2RAs (Figure 4C). Results showed that the addition of dTat-BK to the extracellular medium does partly rescue cells from inhibition of proliferation induced by FR173657 or NG68. This would suggest a possible competition for receptor binding between these two agents at intracellular locations, most likely the nucleus. Similar anti-proliferative effects of CP-B2RAs (at 10 µM), but not of HOE 140, were observed in anchorage-independent growth (Figure 4D) and common MTT assays (Figure 5), adding further support for an inhibitory effect of CP-B2RAs on TNBC growth. As it was observed in clonogenic assays (Figure 4A), the addition of BK alone at high concentration (10 µM) to MDA-MD-231 cells did not induce cell proliferation according to metabolic MTT assays (data not shown).

### CP-B2RAs induced apoptotic death and cell cycle arrest of MDA-MB-231 cells

**Figure 6:**
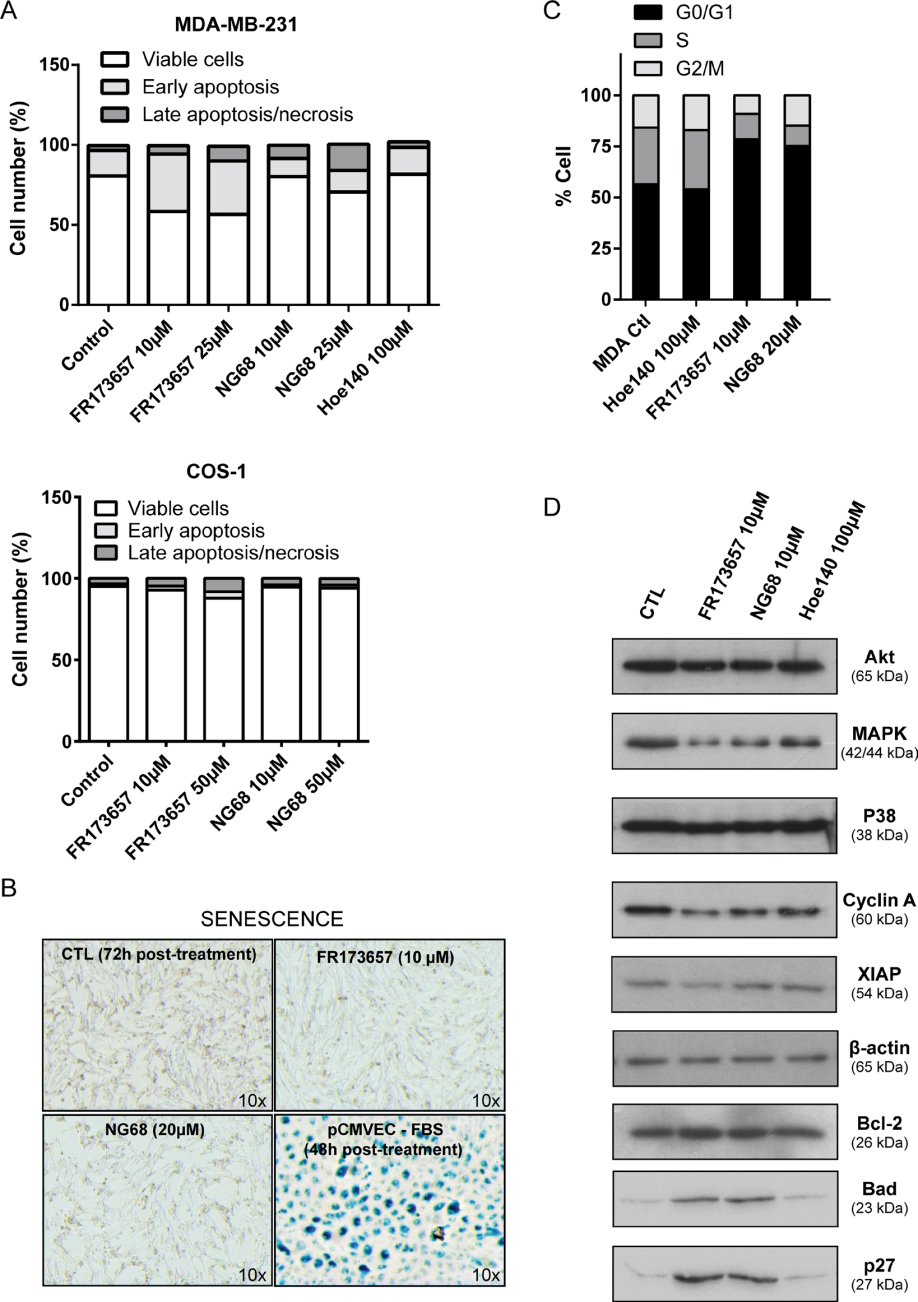
CP-B2RAs induce apoptotic death and cell cycle arrest of MDA-MB-231 cells (**A**) Effects of B2RAs on apoptosis induction were investigated by Annexin V-FITC/ propidium iodide (PI) double staining followed by flow cytometry. Confluent cells were treated with the B2RAs at the indicated concentrations for 24h. Each bar represents mean values acquired from two independent experiments. (**B**) Effects of B2RAs on cellular senescence were analyzed by β-galactosidase staining following a 72h treatment. The pictures show representative contrast phase images of senescence-associated β-galactosidase expressing cells at 10x magnification. Serum-starved porcine cerebral microvascular endothelial cells (pCMVEC) served as a positive control for senescence. A repetition of the experiment gave similar results. (**C**) Effects of B2RAs on cell cycle were evaluated by flow cytometry of cells stained with PI. Each bar represents mean values acquired from two independent experiments. (**D**) Changed expression levels of cell cycle and apoptosis related proteins were evaluated after a 24h-treatment with B2RAs using Western blot analysis. Whole-cell lysates were blotted for the indicated proteins with the appropriate antibodies. Representative western blot of two independent experiments performed.

Beside the inhibition of proliferation and clonogenicity, we evaluated whether the two most potent CP-B2RAs, FR173657 and NG68, may also disrupt other cancer processes as maintenance of cell viability (through activation of either apoptosis or senescence program) and cell cycle progression (Figure 6). We demonstrated that MDA-MB-231 cells underwent apoptosis, but not senescence, as a result of CP-B2RA treatments, as evidenced by the Annexin V and active caspase-3 positivity (Figure 6A) and the lack of expression of β-galactosidase, a widely used senescence biomarker (Figure 6B). In contrast, B2R non-expressing COS-1 cells were only minimally affected when exposed to high concentrations of CP-B2RAs (Figure 6A), indicating a good degree of specificity of these antagonists for B2R target. Control experiments showed that vector dTat alone did not exhibit intrinsic activity (up to 100 μM) in any of the above assays, as expected from literature precedent [40]. We also found that both FR173657 and NG68 tend to induce G0/G1 cell cycle arrest (Figure 6C), which could be one of the primary mechanism for the observed antiproliferative effects of these compounds. In contrast, HOE 140 (up to 100 μM) had no influence on cell cycle distribution of MDA-MB-231 cells (Figure 6C). Western analysis of selected proteins revealed that the CP-B2RAs NG68 and FR173657 may exert antiproliferative and apoptosis-inducing activities through both negative and positive regulatory effects of important proteins involved in different aspects of apoptosis and the cell cycle (Figure 6D). For instance, both CP-B2RAs decreased the levels of MAPK and cyclin A, equally known to be overexpressed and causally implicated in the hyperproliferation of BC [41, 42]. FR173657 also appeared to reduce the levels of the anti-apoptotic protein XIAP. On the other hand, both CP-B2RAs increased expression of pro-apoptotic BAD and of the potent cell cycle inhibitor p27^Kip1^ in MDA-MB-231 cells (Figure 6D). While the exact nature of these changes in protein expression remains to be fully understood, they stand compatible with a clear anticancer effect of the CP-B2RAs.

### P38 MAPK signaling pathways contribute to the antigrowth activities of CP-B2RAs

**Figure 7:**
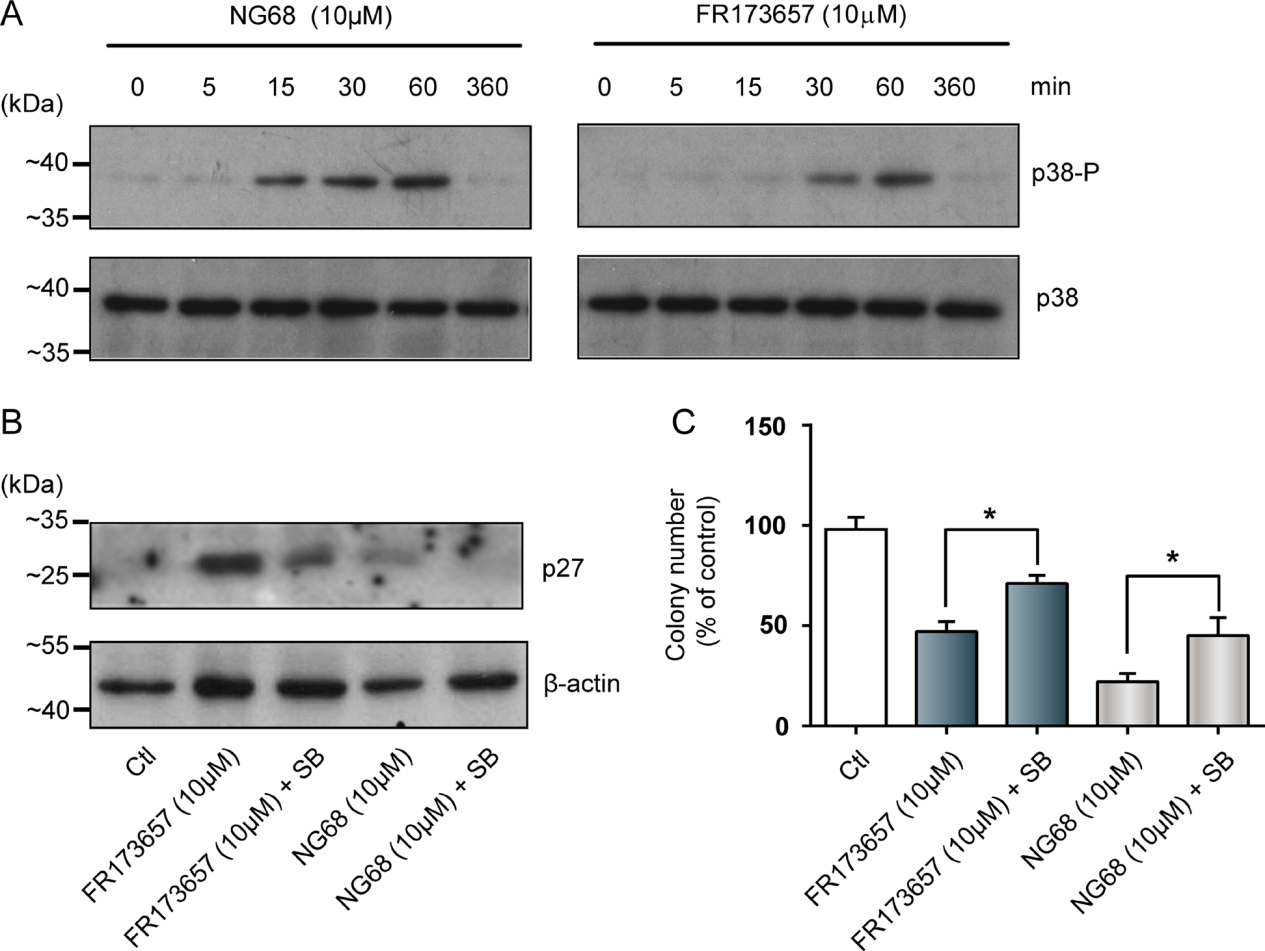
CP-B2RAs promote increased levels of the cell-cycle inhibitor p27kip and reduce clonogenic growth through p-38-dependent pathways Effects of NG68 and FR173657 on (**A**) the phosphorylation (and activity) status of MAPK p38, and (**B**) the expression of p27kip1. To detect the phosphorylation of p38, cells were treated with or without CP-B2RAs for the indicated times. To determine p27 expression, cells were treated with the same antagonists at indicated concentrations for 24h. Whole cell lysates were analyzed by western blotting using the indicated antibodies. β-actin was used as an internal loading control in each case. Representative autoradiograms of 2–3 experiments. Also added in panel B are the effects of the MAPK p38 inhibitor SB203580 (SB) on FR173657 and NG68-triggered expressions of p27kip1. Cells pretreated for 6h with SB (10 µM) were treated with CP-B2RAs at indicated concentrations for 24h. Whole cell lysates were prepared and analyzed by Western blot as in A. (**C**) Effects of MAPK p38 inhibitor, SB203580 on FR173657- and NG68-induced anti-clonogenic activities against MDA-MB-231 cells. Cells pretreated for 6h with SB (10 µM) were treated with or without CP-B2RAs at indicated concentrations for 14 days. Clonogenecity efficiency was evaluated as described in Figure 3; SB alone had no effect (not shown). Data are as mean ± s.e.m. of 6–8 experiments. Ctl, control (no treatment). ^*^p < 0.05 versus the indicated group, using Student’s *t*-test for unpaired samples.

It was shown that p38 MAPK, in particular p38α, can act as a tumor suppressor by inducing G1 cell-cycle arrest and apoptosis [43]. We therefore focused our attention on the p38-signaling pathway cascade and tested whether it is involved in the antiproliferative and pro-apoptotic activities of CP-B2RAs (Figure 7). Results of western blot assays showed that the both FR173657 and NG68, significantly increased the phosphorylation (and resultant activity) of p38 over time (Figure 7A). Moreover, pretreatment of MDA-MB-231 cells with the p38 MAPK inhibitor SB203580 (10 µM) attenuated the CP-B2RA-induced increases of p27/Kip1 protein levels (Figure 7B) as well as their anti-proliferative activities in clonogenic assays (Figure 7C).

### shRNA-knockdown of B2R suppressed basal growth and antiproliferative activities of CP-B2RAs *in vitro*

**Figure 8:**
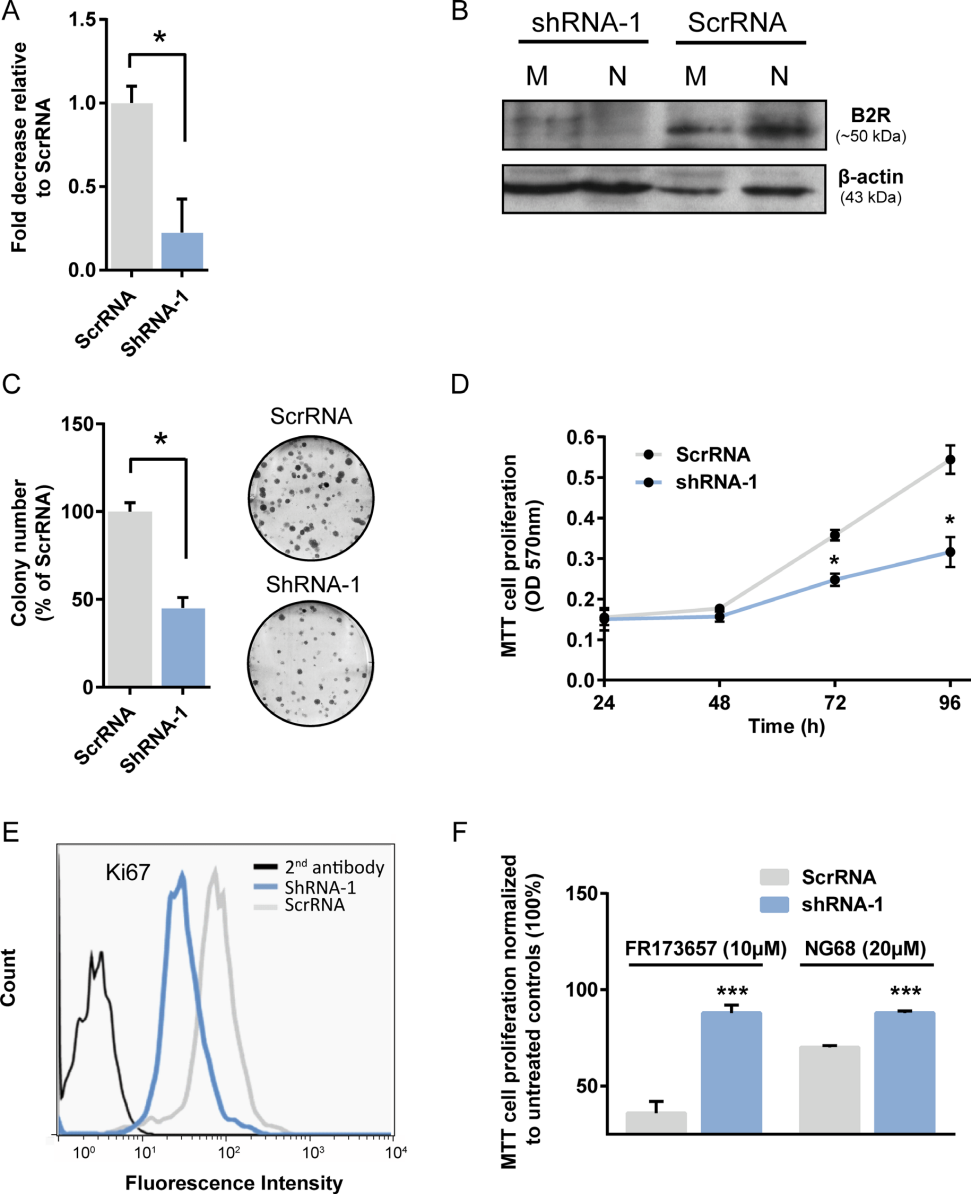
Stable B2R knockdown affects basal growth and cytocidal activity of CP-B2RAs in MDA-MB-231 cells (**A**–**B**) Knockdown efficiency of B2R was assessed using quantitative real-time PCR and western blot analyses. (**A**) Graph indicates the mean ± s.e.m. from 3 independent experiments. (B) The autoradiograms shown are representative of two experiments. (**C**–**D**) Effects of B2R depletion on clonogenic and proliferative potential of MDA-MB-231 cells under basal conditions. Clonogenicity and MTT cell proliferation experiments performed as described in Figures 3 and 4, and under Materials. (C) Graph indicates the mean ± s.e.m. from 3 independent experiments. Representative images of methylene blue-stained colonies of MDA-MB-231 cells expressing B2R shRNA or a scrambled shRNA are shown on the right side. (D) Data are as mean ± s.e.m. of 6–8 experiments. (**E**) Altered expression of the proliferation marker Ki67 in MDA-MB-231 B2R knockdown cells measured by FACS. Data are representative of two independent experiments. (**F**) Effects of B2R depletion on the cytostatic activities of CP-B2RAs against MDA-MB-231 cells determined by MTT assays. Data are as mean ± s.e.m. of 6–8 experiments. Basal viability of ScrRNA was comparable to that of shRNA-1 (not shown). All statistical analyses were performed using Student’s *t*-test for unpaired samples. ^*^p < 0.05, ^***^p < 0.001 versus the indicated group or corresponding time points.

To determine whether B2R participated in the proliferative and clonogenic capacities of MDA-MB-231, and to further prove that the anticancer effects of CP-B2RAs were not biased by unsuspected off-target effects, MDA-MB-231 cells were stably transduced with lentivirus expressing encoding either scrambled control sequence or shRNA targeting B2R (Figure 8). Knockdown efficiency of B2R was determined by qPCR and Western blot analyses. We found that both B2R mRNA and protein levels were reduced by up to 75–80% in the B2R-silenced MDA-MB-231 cells compared to scrambled control shRNA-treated cells (Figure 8A, 8B). Notably, depletion of B2R proteins was observed to the same extent at both nuclear and plasma membrane compartments as detected by Western blot (Figure 8B). Having validated the shRNA-mediated gene silencing of B2R, we next investigated the effect of B2R knockdown on cell proliferation using clonogenic and MTT assays along with immunostaining of the proliferation marker protein Ki67 estimated by FACS (Figure 8C, 8D, 8E). We noted a marked, profound inhibition of proliferation and clonal growth (by more than 50%) of TNBC cells that had been depleted of B2R (Figure 8C, 8D). B2R knockdown MDA-MB-231 cells also demonstrated decreased levels of Ki67, confirming the intrinsic proliferation defect in these cells (Figure 8E). These results clearly indicate the involvement of B2R in the regulation of TNBC growth under basal (unstimulated) state.

Importantly, B2R downregulation in MDA-MB-231 cells significantly attenuated the cytostatic activities of both CP-B2RAs, FR173657 and NG68 (Figure 8F). These results and other also presented herein are congruent with the possibility that CP-B2RAs exert their anticancer action at least partly via intracellular-specific functional B2R, most likely located at the nucleus.

### Comparison of *in vitro* cytotoxic activities of CP-B2RAs with standard chemotherapeutic agents currently in use for the treatment of TNBC

To highlight the importance of intracellular B2R in TNBC, we compared the relative effectiveness of CP-B2RAs with that of standard chemotherapies in inducing TNBC cell death by using MTT viability assays (Table 2). Chemotherapeutic drugs (*e.g.,* BCNU, doxorubicin, carboplatin, temozolomide and paclitaxel) approved by the FDA for the treatment of TNBC were included in this analysis [3]. Anticancer potencies (expressed as mean IC_50_ values (µM)) of all tested agents are summarized in Table 2. Both CP-B2RAs, FR173657 and NG68, showed strong anticancer potencies which were however slightly inferior (i.e. higher IC_50_ values) to those of doxorubicin and paclitaxel. On the other hand, both CP-B2RAs were considerably more potent than the three DNA alkylating agents BCNU, carboplatin, and temozolomide. In fact, carboplatin and temozolomide did not show significant cytotoxic effect even at a concentration of 100 µM. Lack of cytocidal activity of alkylating agents may not come as a surprise as MDA-MB-231 cells show the highest level of resistance to an array of chemotherapeutic drugs by comparison to other BC cancer lines (*e.g.* MDA-MB-435, MDA-MB-468, MDA-MB-453S, and MCF-7), which is most likely due to MDR and P-gp overexpression and function [44].

**Table 2:** Cytocidal activities of B2RAs and standard chemotherapeutic agents against MDA-MB-231 cells

Agents	IC_50_ values
HOE 140	> 100 μM
FR173657	20 ± 1 μM
NG68	45 ± 1 μM
NG134	> 100 μM
BCNU (carmustine)	62 ± 1 μM
Adriamycin (doxorubicin)	2 ± 1 μM
Carboplatin	> 100 μM
Temozolomide (Temodar)	> 100 μM
Paclitaxel (Taxol)	10 ± 1 μM

Data represent means ± s.e.m. (*n *= 6 to 8 experiments). Cell viability was established by the MTT assay after 72h drug exposure. IC_50_ values were calculated from complete concentration response-curve from no response to 80–100% cell death.

To assess whether a combination of a CP-B2RA with a chemotherapeutic agent may provide a mutual enhancement of the therapeutic effects, pair-wise combinations of these drugs (through IC_25_ values) were analyzed in MDA-MB-231 cells, and cell viability inhibition was assessed as described earlier. The CalcuSyn program was then used to calculate combination indices (CI) for the two anticancer drugs. We found that the FR173657 and doxorubicin or paclitaxel combinations resulted in augmented killing of TNBC cells in a synergistic manner, as reflected by CI values well below 1. TNBC cell death was also augmented by NG68/paclitaxel or NG68/doxorubicin combinations, being additive (CI close to 1) to moderately synergistic (CI < 1), respectively. Thus, these results suggest that the combination of CP-B2RA and chemotherapeutic agents may provide a convenient means to optimize treatment of TNBC.

## DISCUSSION

To elucidate the involvement of internal/nuclear GPCR, more specifically B2R, in TNBC tumorogenesis, we designed and/or characterized newly kinin CP-B2RAs that were tested as anticancer agents. Here we present novel data demonstrating 1) strong constitutive intracellular/nuclear B2R localization in TNBC cells and tissue specimens; 2) that CP-B2RAs can interact with nuclear B2R sites and exhibit potent anticancer activities in cultured TNBC cells; a common mechanism underlying their anticancer effects may involve induction of G1-phase growth arrest and apoptosis through activation of p38kinase/p27^Kip1^ pathways; and 3) that targeting intracellular B2R using CP-B2RAs is more effective than currently utilized chemotherapy (*e.g.,* carboplatin, temozolomide) while acting synergistically or additively with others (*e.g*., doxorubicin or paclitaxel) to enhance their mutual cytocidal effects against TNBC cells.

The exact mechanism explaining the strong nuclear localization of B2R in TNBC cells/tissues remains unclear. Previous work by Lee *et al.* has led to the identification of a putative nuclear localization signal (NLS; motif K-K/R-X-K/R) at the C-terminus of human B2R [45], which could contribute to explain the latter observation. In keeping with this strong possibility, several studies using deletion/truncation NLS mutagenesis experiments have demonstrated the importance of an intact NLS for the nuclear localization or translocation and physiologic functions of several GPCRs, including the leukotriene CysLT1 receptor [12], angiotensin AT1 receptor [46], parathyroid hormone type 1 receptor [47], gonadotropin releasing hormone type 1 receptor [48], chemokine receptor CXCR4 [18], orphan receptor GPR158 [49], formyl-peptide receptor 2 [50] and the platelet-activating factor receptor [51]. Therefore, it is most likely that the nuclear import of B2R in TNBC cells occurs along this redundant pathway. We do not however rule out the contribution of other recently described alternate pathways involving small GTPases and sorting nexin proteins [23]. Our new findings, which describe the aberrant nuclear localization of B2R (via whatever mechanism) in TNBC are consistent with the recent work of Takano *et al.* demonstrating that B2R is able to form heterodimers with lamin C in the cell nucleus independent of BK stimulation [52].

It should be noted that, although our data showed high nuclear localization of B2R and antineoplastic activities of the CP-B2RAs in MDA-MB-231 cells, these two phenomena may not be directly or strictly linked. Interaction of CP-B2RAs at subcellular sites other than the nuclear compartment may possibly exist for the CP-B2R to initiate intracellular signaling pathways contributing to their antiproliferative and cytocidal activities. Such inference is supported by many studies, though not related to the field of BC, describing the presence of functional native GPCRs at various organelles, such as mitochondria (*e.g*., angiotensin AT1R, cannabinoid CB1R), endosomes (*e.g*., adrenergic β2R, BK-B2R), lysosomes (*e.g.,* cannabinoid CB1R, ocular albinism OA1R or GPR143), endolysosomes (*e.g.*, L-α-lysophosphatidylinositol GPR55) and the endoplasmic reticulum (*e.g*., estrogen GPR30, vasopressin V2R) [53–56]. Other studies will be required to address this possibility.

We, our collaborators and other colleagues, have demonstrated the existence and function of several GPCRs (*e.g*., PGE2/EP3, PAFR, LPA1R, AT1R, B2R, ApelinR, GnRHR, and trypsin/PAR2) along with their signal transducing components (*e.g.,* Akt, MAPK p42/p44, eNOS, and COX-2) at the cell nucleus of multiple cell types (see reviews [10, 19, 23, 57, 58]). These work has revealed new roles for these GPCRs in regulating nucleoplasmic calcium and ensuing expression of diverse target genes that are involved in inflammation and cell proliferation/growth [10, 19, 23, 33, 57, 59]. Importantly, these nuclear GPCRs can function in ways distinct from its plasma membrane counterpart. So far, however, most evidence to ascribe unequivocally the functional significance of nuclear GPCRs for peptides were obtained using *in organello* nuclear assays on intact nuclei isolated from cells and tissues under normal and pathological conditions (see reviews [10, 60]). Despite their obvious and substantial utility for introducing this new concept, there remains a definite need to validate the functions of nuclear GPCRs in living cells or organisms, and perharps even more so, in cancer pathology in which mitogenic signals may arise from persistent and excessive activity of nuclearly overexpressed GPCRs. With this in mind, we created novel molecule internalizing-vectors that can promote the permeation and incorporation of hydrophilic drugs as the potent peptide B2RA HOE 140 (Table 1), thus providing prototypic strategies to target intracellular/nuclear GPCRs. Our results showed that our conjugation approach has succeeded in fulfilling the task and revealed that stabilized dTat is more effective than steroidal vector at delivering HOE 140 across the plasma membrane of MDA-MB-231 cells. Our results also established for the first time, to our knowledge, the cell-penetrating property of the non-peptide B2RA FR173657. Moreover, all of the newly-described CP-B2RAs turned out to be potent repressor of TNBC growth, mostly dependent of p38 MAPK signaling. FR173657 was particularly effective in this regard. Its relative tendency to accumulate at much higher concentration than the other CP-B2RAs into tumor cells may explain the greater anticancer potency of the drug (Table 1). Such differences in the magnitude and rate of uptake between CP-B2RAs may reflect divergent paths of cellular entry and/or degradation after entering the cells. For instances, cellular uptake of lipophilic agent, as FR173657, can occur via passive membrane penetration while that of cationic Tat-peptides, as NG68, is believed to take place mostly by macropinocytosis [42].

Supplementary data on additional signal transduction pathways associated with the anticancer effects of FR173657 on MDA-MB-231 cells, determined through microarray gene-profiling studies, can be found online (see Table S1). It is conceivable that the important antitumor effects of FR173657 will be shared by other structurally-related, lipophilic non-peptide B2RAs, for example, the compound MEN16132 [61] or JSM10292 [62]. Yet consistent with the idea of a possible intracrine action of B2RAs, is the strong and complex cytotoxic effects reported with the small non peptide B2RA mimetic BKM570 on various human cancer cell lines, which are apparently mediated independently of cell-surface B2R functions [63, 64]. Furthermore, previous studies showed that the peptide B2RA B-9870 (CU201) can inhibit growth of breast tumor cells [31, 65], within the same concentration range of the CP-B2RAs used herein. Likewise NG68, B-9870 is an Arg-rich cationic peptide containing four Arg and two d-Arg. It is therefore possible that B-9870 can harbor the same effective cell membrane transduction property as that of NG68, allowing it to cross plasma membrane to reach intracellular space and intended target, as initially postulated by Morissette et al. [31]. It is interesting to note that, in our study, HOE 140 was found inactive (up to 100 µM) as an anticancer drug while other *in vitro* studies have demonstrated that it can act as a potent mitogenic agonist [66] or, conversely, as a antiproliferative and apoptotic agents [67], albeit using cancer cell lines of other origins.

Although alternative interpretations remain possible, the fact that the CPB2RAs still remained fully active even after having previously blocked cell-surface B2R with excess of HOE140 (10 µM) (Figure 4) and had reduced cytostatic activities in partly B2R depleted-TNBC cells (Figure 8), are highly suggestive signs of an intracrine mechanism at work.

The mechanisms by which BK induces BC cell proliferation following activation of cell-surface B2R are not completely defined but may at least involve activation of the classical ERK/MAPK pathway [7, 25]. Recent seminal work by the group of Ehrenfeld *et al.* has shown that the kinin-generating kallikrein KLK1 and its substrates, kininogens, are expressed in estrogen-sensitive (MCF-7, T47D, ZR-75–1) and estrogen-insensitive (MDA-MB-231) BC cells [68]. In the present study, we managed to independently reproduce these results by immunolabeling and confocal microscopy (Supplementary Figure 3). We indeed detected the presence of endogenous kininogens-, kallikreins- and BK-like immunoreactivities in MDA-MB-231 cells not only in the cytoplasmic region, but also in the intranuclear region of MDA-MB-231 cells (Supplementary Figure 3), which were generally characterized by a fine granular speckled cytoplasmic and nuclear staining. Such kinin-generating system was also reported to be present in the human glioblastoma cell line U-373 [69]. Overall, these results give credence to the possibility that the tumor-derived BK may be active both intracellularly (at nuclear sites) and extracellularly (upon its release) on cancer and stromal cells, thereby contributing to tumor growth and progression. The same tenet has also been postulated in the regulation of alternative functions of VPAC1 receptors depending on their spatial distribution in the development of breast cancers [20]. Future research is needed to shed more light on the biochemical mechanisms involved in the regulation of BK formation and circumscribed release either inside or outside cancer cells, allowing BK to operate via intracrine, autocrine or paracrine mechanisms.

Several strategies have been used recently for dissecting membrane and nuclear effects of peptide hormones interacting with GPCRs in intact living cells. Among others, there are the use of cell permeabilization by pore-forming toxins or detergents and ligand intracellular microinjection [70–72]. While, there may be some concerns as to whether semi-intact cells reflect a genuine and valid model for intact living cells, both techniques may result in significant impediments to membrane integrity and cytoskeletal structure, leading to perturbation of cell functions [73, 74]. A new interesting approach has recently been developed to selectively study the intracrine signaling of peptide ligands (*e.g.,* angiotensin and endothelin) via intracellular/nuclear GPCR, which consists of cell-permeant, photorelasable, caged ligands [75, 76]. The general principle is that, once the functionaly inert, caged ligand gets inside the cell, the ligand is released from the cage by exposing cells to UV lights, allowing it to interact and modulate functions of its cognate intracellular receptors. However, application of this approach *in vivo*, most notably in the context of cancer therapy (excluding skin cancer), may not be yet practically feasible since UV radiations do not penetrate the skin very deeply. Hence, should our results be validated *in vivo* in recognized animal models, we believe that the application of molecule-internalizing vectors as those herein and others described in the literature [36, 74] to enhance permeation of GPCR targeting-drugs through cancer cells (if needed) should be actively pursued in therapeutic cancer setting.

In conclusion, our data support the existence of an intracrine signaling pathway via internal/nuclear B2R that appears critical for growth of TNBC cells. We engineered new approaches and discovered new tools to target intracellular B2R relevant for TNBC. Thus, our research has resulted in defining a new class of B2RAs to overcome the limited efficacy of current strategies in tackling TNBC. In sum, the results highlight the need to target a given GPCR in relation to its subcellular localization in order to achieve therapeutic outcomes.

## MATERIALS AND METHODS

### Human tissue samples and electron immunohistochemistry of B2R

Human triple-negative invasive ductal carcinoma (IDC) samples and matched normal adjacent tissue specimens were obtained from two patients who underwent surgical resection of their primary tumor at the Hôtel-Dieu Hospital in Sherbrooke (Québec, Canada). The use of the tissue samples was approved by the Institutional Review Board. Post-embedding immunogold labeling of B2R was performed on ultrathin Epon sections (50 nm) mounted on nickel grids as described [33, 77]. Sections were incubated with the rabbit anti-rat (cross-reacting to human) B2R antiserum AS-276–83 (1:25) followed by incubation with a goat anti-rabbit gold (10 nm)-conjugated IgG (1:20) (Sigma Chemical Co.). Sections were contrasted, embedded and viewed as described [77]. Negative controls were carried out by omitting primary antibodies, replacing it with preimmune serum or an isotype control IgG. The areas of nuclear and cytoplasmic portions were measured by using Image J software (National Institute of Health, JAVA 1.60_02) [78]. An average of 10 to 15 cells covering an area of at least 200 µm^2^ per case were examined, and each case generated about 50 images of magnifications ranging from 40,000x to 60,000x. A total area of 1,904 µm^2^ was examined in normal or cancer cells. Parameters used for statistical comparisons were 1) the number of gold particles in the nucleus/ nucleus area and 4) the number of gold particles in the cytoplasm/ cytoplasmic area.

### Solid phase peptide synthesis

All peptides were assembled on solid support by an automated Pioneer peptide synthesizer using Fmoc (9-fluorenylmethyoxy-carbonyl) chemistry [34]. Peptides were purified by analytical RP-HPLC and were > 95% pure, with expected mass spectra (see supplementary Methods Supplementary Figure 4 for details on purification and identification of peptides used in this study). As part of a project that aimed to design new cell-penetrating peptides, we synthesized a series of cell-penetrating kinin peptides by linking the modified HIV-1 Tat peptide sequence H-^48^GRKKRRQRRR^58^-OH in d-configuration (to confer proteolytic stability) to the N-side of BK and HOE 140 sequence terminated with 3 glycyl residues as a spacer (for steric hindrance considerations). Another approach consisted of conjugating the steroidal moiety cholic acid to the N-terminal part of the peptide HOE 140 linked to the resin. Fluorescein isothiocyanate (FITC) was linked to a β-alanine residue added to the N-teminus of the peptide HOE 140 and dTat-HOE 140 still attached to the resin. Peptides were stored in powder form at – 20°C. Stock solutions (10 mM) of peptides were also prepared in Nanopure water, except for the steroid peptide conjugate, which was prepared in 50% ethanol-50% Nanopure water, and then stored at – 20°C until use. The non-peptide B2R antagonist FR173657 was provided by Astellas Pharma Inc. (formelly Fujisawa Pharmaceutical Co, Japan) [79] and was prepared in 50% dimethyl sulfoxide (DMSO)-50% Nanopure water.

### Distribution coefficient of compounds

The distribution coefficient of compounds (log D) between n-octanol and 0.1 M sodium phosphate buffer, pH 7.4, was determined by the classical shake flask methodology; this parameter is thought to be a good predictor of the cell membrane permeability. Equal volumes of octanol and PBS (137 mM NaCl, 2.7 mM KCl, 10 mM Na_2_HPO_4_, 2 mM KH_2_PO_4_, pH 7.4), were mixed and allowed to equilibrate for 24h. The layers were then separated and stored at 4°C. At testing, the compounds (200 µM) was added to 200 µl of aqueous phase and mixed with an equal volume of octanol by vortexing for 2 min. The octanol/buffer solution was centrifuged for 5 min at 1000 x g. After separation into aqueous and octanol phases, compound content of the aqueous and octanol phases was quantified by RP-HPLC (Agilent1100 series). Partition coefficients of compounds were expressed as the ratio of peptide found in the octanol phase to that found in the aqueous phase. All octanol/buffer distribution studies were performed in duplicate. The octanol/buffer coefficient was calculated as the ratio of octanol layer to the buffer layer. Compounds with log D values higher than 0 and less than 3 fall into the highly permeable class.

### Binding assays

Radioligand competition binding assays were performed on live adherent HEK-293T cells transiently transfected with human B2R using [^3^H]LysdesArg^9^-BK, according to Bélanger *et al* [34]. Binding affinity of the compounds is expressed in terms of IC_50_ value: the molar concentration of an unlabeled agonist causing 50% displacement of specific binding. Radioligand competition binding assays was also conducted on MDA-MB-231 nuclear extracts using ^125^I-hpp-HOE 140 prepared as described previously [80]. Briefly, binding assays was done in presence of 0.5 nM ^125^I-hpp-HOE 140 with or without 1 µM HOE 140 (B2R selective) or 1 µM R954 (B1R selective) in a buffer solution of 50 mM Tris-HCl, pH 7.4, 10 mM MgCl_2_, 10 µM captopril and 10 µM thiorphan. Reaction was initiated by adding 200 µg of nuclear protein extracts and the incubation conducted at room temperature for 1h. Each measurement was performed in triplicate in a final assay volume of 100 µl. This incubation was terminated by rapid filtration through GF/C filters (Whatman) soaked in 0.3% (vol/vol) polyethyleimine/binding buffer. Cell nuclear bound radioactivity was measured using a γ-radiation counter (WIZARD2, Perkin Elmer).

### Functional bioassays with isolated human vessels

For testing the pharmacological activities of agonists, complete concentration-contractile response curves to agents were done at the B2R endogenously expressed in the human de-endothelized umbilical veins (hUV), as previously described [34]. EC_50_ values (or potencies) of the agonists were calculated from the obtained concentration-response curves. Antagonist apparent affinity is expressed in term of IC_50_ value: the molar concentration of an antagonist that reduces a specified BK submaximal response to 50% of its former value. The collection of umbilical cords for the bioassays was approved by the local ethical committee, and maternal consent was obtained in every case.

### Cell culture and reagents

The human breast cancer cell line MDA-MB-231 and COS-1 monkey kidney epithelial cells were maintained in DMEM medium containing 10% heat inactivated fetal bovine serum (FBS), 2 mM L-glutamine, 100 U mL^−1^ penicillin and 100 µg/mL streptomycin. The primary culture of cerebral microvascular endothelial cells from piglets (courtesy of Dr. S. Chemtob, Canada) was cultured in DMEM medium as described [81, 82]. Cells were treated as described in figure legends or specific method section.

Antibodies were obtained from the following sources: anti-clathrin antibody, anti-Na+/K+ ATPase antibody, anti-lamin A/C antibody, anti-Bcl-2 antibody, anti-cyclin A antibody, anti-p27 antibody (Santa Cruz Biotechnology); anti-β-actin antibody (Sigma-Aldrich); anti-MAPK p42/p44 antibody (Promega); anti-XIAP antibody (Stressgen); anti-calnexin antibody, anti-Ki67 antibody (Abcam); anti-Akt antibody (New England BioLabs); anti-p38 antibody (Chemicon international); anti phospho-p38 antibody (Millipore); anti-hB2R antibody (C-terminus) (LS-A797: LifeSpan Biosciences); anti-LAMP-2 antibody (ALZForum); anti-GBM-130 antibody (BD Transduction Laboratories); anti-Bad antibody (BD Biosciences); rabbit polyclonal Isotype control antibody (Dako). The rabbit anti-human B2R antisera AS276–83 (targeting 8 distinct epitopes on the extra–and intracellular domains of B2R) and the mouse monoclonal antibody anti-human kininogens (directed to domain D1) were provided by Pr W. Muller-Esterl (University Frankfurt) whereas the rabbit polyclonal anti-tissue kallikreins and anti-BK antisera were provided by Prs F Alhenc-Gelas and N Bouby (INSERM). Chemotherapeutic agents were obtained from the following suppliers: carmustine (or BCNU) and paclitaxel (Taxol) (Sigma-Aldrich); doxorubicin (Adriamycin), carboplatin (Paraplatin), temozolomide (Temodar) (pharmacy department of the Centre Hospitalier Universitaire de Sherbrooke (CHUS)).

### Generation of lentiviruses and infection

Custom non-target (scrambled control) and human B2R targeting lentiviral short hairpin RNA (shRNA) expression-pLKO.1 vectors were purchased from Sigma-Aldrich. The non-targeted and targeted sequences were as follows: shRNA scrambled: 5′CCTAAGGTTAAGTCGCCCTCGCTCGAGC GAGGGCGACTTAACCTTAGG3′ (Addgene plasmid # 1864) shRNA-B2R: 5′CCGGCGAGCGCATCATCGATGTAATCTCGAGATTACATCGATGATGCGCTCGTTTTT3′ (designated as shRNA-1). The shRNA lentiviruses production in HEK-293T cells and the infection of MDA-MB-231 cells with the viral suspension were performed according to manufacturer’s instructions (Sigma-Aldrich). MDA-MB-231 cells stably infected with lentiviruses encoding shRNA-B2R or scrambled shRNA sequence were obtained after a 10 day selection with puromycin (2 μg/mL).

### Real-time quantitative RT-PCR

Total cell RNA was isolated with the RNeasy mini kit (Qiagen, Toronto, ON, Canada) according to the manufacturer’s protocol. Reverse transcription was performed at 42°C for 50 min, using SuperScript II reverse transcriptase and random hexamer primers (Invitrogen). Quantitative PCR was performed on an M3000p Stratagene instrument (Agilent technologies), 40 cycles of 95°C for 15s and 60°C for 30s using SYBR Green (Quanta Biosciences). The following primers were used: for B2R (NM_000623.3), 5′-AGTACCAGGGAGCGACTGAA-3′ (forward) and 5′-GTGACATTGAGCATGTCGGC-3′ (reverse) (amplicon length, 176 bp); for β-actin (NM_001101.3), 5′-GTTGCTATCCAGGCTGTGCTA-3′ (forward) and 5′-GCGGATGTCCACGTCACACTT-3′ (reverse) (amplicon length, 471 bp). The comparative Ct method was used to quantify transcripts that were normalized with respect to β-actin expression. Control reactions were performed in the absence of reverse transcriptase to demonstrate that residual contaminating DNA is not present in our RNA preparation.

### Clonogenic assay

MDA-MB-231 cells were plated at 200 cells/well in a six-well dish in 2 ml DMEM medium containing 10% FBS. For establishing concentration-response curves, cells were treated with either B2R agonists (BK and dTat-BK) or antagonists (HOE 140, dTat-HOE 140 (NG68), Cholic-HOE 140 (NG134) or FR173657) at concentrations ranging from 1 to 100 µM. In some experiments, cells were pre-exposed to HOE 140 10 µM (for obstructing cell-surface B2R) for 15 min, and then incubated with the other tested B2R antagonists (NG68, NG134 or FR173657). After 10 to 14 days, colonies were fixed and stained with 0.5% methylene blue in 50% ethanol. Excess of staining solution was removed carefully with tap water and the plates were dried overnight. Colony counting and densitometric analyses (to measure total colony numbers and surface areas) were performed using the Image Pro Plus 5.1 software.

### Anchorage-independent growth (soft agar assay)

MDA-MB-231 cells were seeded out into 6-well plates (at 6,000 cells per well) and cultured in DMEM media with 10% FBS. The following day, cells were treated with and without the B2RAs at the indicated concentrations for one week. At the end of treatment, about 3,000 viable cells were resuspended in 3 mL of DMEM medium containing 10% FBS. Molten agar (about 42°C) was added to each tube containing the cell suspension, and the agar-cell mixture was promptly put into wells of a 6-well plate. To avoid drying of agar, 1 mL of DMEM (10% FBS) was added to each well, and renewed every few days. Cells were incubated at 37°C for about 3 weeks. Colonies were counted by two persons blindly (AF, VB) without any form of staining using an inverted microscope.

### Cellular fractionation

Cell fractionation and nuclei isolation were achieved by the hypotonic/Nonidet P-40 lysis methods [82] with slight modifications. Briefly, cells were harvested at confluency, washed in pre-warmed HBSS then resuspended in hypotonic cell lysis buffer (20 mM Tris-HCl pH 7.2, 10 mM KCl, 3 mM MgCl_2_, with protease inhibitors). Cell were left to swell on ice for an hour and then lysed in presence of NP40 (0.1% vol/vol) using a Dounce homogenizer (300 strokes, tight pestle). Nuclei were washed twice (7 min at 800g, 4°C) in lysis buffer and nuclei purity was verified under phase contrast microscope. The supernatant from the first centrifugation of cell lysates was sequentially centrifuged at 10,000g, 4°C for 15 min (to pellet mitochondria), and then at 200,000 g, 4°C for 60 min (to pellet membranes). Membranes were resuspended in Phosphate buffer containing protease inhibitors. Total proteins in cell lysates, nuclear and membrane suspensions were measured using BCA™ (bicinchoninic acid) protein assay kit (Pierce).

### Western blot analysis

For western blot analysis, appropriate amount of cell lysates or membrane and nuclear fractions (25–50 µg protein) were denatured, resolved on 9–12% SDS–PAGE and electrotransferred onto PVDF membranes. Blots were blocked using 5% nonfat dry milk and probed using appropriate primary antibodies in blocking buffer overnight at 4°C. Proteins were visualized using a goat anti-rabbit (1:10 000) or anti-mouse (1:20 000) secondary antibody conjugated to HRP and a western lightning chemiluminescence detection kit, as per manufacturer’s instructions (Perkin Elmer), and revealed by use of Kodak X-Omat film.

### MTT proliferation and cytotoxicity assays

Cell proliferation assay on MDA-MB-231 cells was determined with colorimetric MTT as we described [9]. Briefly, cells were seeded in a 96 wells plate at 2,000 cells/well in DMEM media supplemented with 10% FBS for 24h at 37°C. Cells were then incubated with and without the B2RAs at the indicated concentrations for the time shown. At the end of treatment period, MTT (0.25 mg/ml) was added to the cells and incubated for 1h30 at 37°C. Thereafter, the cell medium was removed, the formazan product solubilized in acidified isopropanol (100 µl of 1N HCl/anhydrous isopropanol *1:25*) and the absorbance determined at 570 nm with an ELISA microplate reader (Spectra Max Plus, Molecular Devices, Sunnyvale, CA). For assessing the cytotoxicity of B2RAs and chemotherapeutic agents (carmustine, doxorubicin, carboplatin, temozolomide and paclitaxel) [83], cells were grown to confluency in multi-well plates and exposed to starving media (DMEM without FBS) for 24h before beginning of the antagonist treatment. Cells were then incubated with MTT and treated exactly as described above. Cell viability was expressed as a percentage of optical density (OD) values obtained upon treatment relative to control.

### Senescence

The effect of B2RAs on cellular senescence was assessed using the Senescence β-Galactosidase Staining Kit (Cell Signaling). MDA-MB-231 cells were plated at 500 000 cells/well in 6-well plates, and serum-starved right before the beginning of treatments. Cells were treated with and without the B2RAs at the indicated concentrations for 72 h. At the end of treatment, surviving cells were fixed with 2% formaldehyde, 0.2% glutaraldehyde for 15 min at room temperature. Fixed cells were then washed and stained with fresh senescence-associated β-galactosidase stain solution (sodium phosphate buffer, pH 6.0, containing 1mg of X-gal (5-bromo-4-chloro-3-indolyl-β-d-galactopyranoside)/ml 40 mM citric acid, 5 mM potassium ferrocyanide, 5 mM potassium ferricyanide, 150 mM NaCl, 2 mM MgCl_2_) at 37°C. Staining was detected by light microscopy. As a positive control for senescence, we serum starved porcine cerebral microvascular endothelial cells (pCMVEC) (kindly provided by S Chemtob) for 48 h.

### Flow cytometry

The subcellular distribution of B2R in MDA-MB-231 cells was determined by flow cytometry (FACS) analysis. MDA-MB-231 cells were detached from culture flasks by accutase treatment, washed in blocking buffer (PBS and 0.5% BSA) with or without 0.1% saponin (for cell permeabilization), and then incubated with the anti-B2R antibody LS-A797 or AS276–83 (1:500) for 60 minutes at 4°C under mild agitation. Thereafter, cells were centrifuged, resuspended in the same blocking buffer (+/- 0.1% saponin) and incubated with the Alexa Fluor 488 goat anti-rabbit IgG antibody conjugate (1:300) under the same condition as set out above. Negative controls included the omission of the primary antibody. A similar protocol was used for B2R staining of isolated nuclei from the same cells. Samples containing cells (or nuclei alone) in suspension were analyzed by flow cytometry (FACScan, BD Biosciences) equipped with a Cell Quest software (BD Biosciences).

### Annexin-V- Propidium iodide (PI) staining (apoptosis)

MDA-MB-231 and COS-1 cells were plated at 500,000/well in 6-well plates containing medium (10% FBS/DMEM), and allowed to adhere for about 18 h. On the next day, cells were serum-starved and then treated with and without the B2RAs at the indicated concentrations for 24 h. To assess induction of apoptosis due to treatment, we used the Annexin-V-FLUOS Staining kit (Roche) according to manufacturer’s specifications. Briefly, at the end of treatments, cells were harvested, rinsed and incubated in 100 µl of Annexin-V-FLUOS labeling solution (containing 2 µl of Annexin-V-Fluos reagent and 1 µl of PI (15 µg/ml)) for 15 min at room temperature. Cells were analyzed on a FACSCanto II flow cytometer (BD Biosciences). Early stage apoptosis was measured using annexin V-Fluos (RocheMolecular Biochemicals) in conjunction with PI (Sigma) to distinguish among early apoptosis and late apoptosis/necrosis. Bar diagram showing the distribution of early, late/necrotic cell populations.

### PI staining (cell cycle)

MDA-MB-231 were plated at 500,000/plate in 10 cm plates containing medium (10% FBS/DMEM), and allowed to adhere for about 18 h. Thereafter, cells were serum-starved and treated with and without the B2RAs at the indicated concentrations for 24 h. At the end of treatments, cells were harvested, rinsed and fixed with ice cold ethanol 95%, for 2 h at 4°C. Fixed cells were rehydrated with 800 µl PBS for 30 min, and then washed again with PBS to remove all traces of ethanol. Cells were treated with RNase for 30 min prior to staining. PI was added to cell suspension at a concentration of 15 µg/ml and incubated overnight at 4°C. PI staining was assessed by flow cytometry using a FACSCanto II flow cytometer (BD Biosciences).

### Ki67 staining (proliferation index)

MDA-MB-231 were plated at 1 × 10^6^ cells/plate in 10 cm plates containing medium (10% FBS/DMEM). 48 h after seeding, cells were washed three times with Hank’s balanced salt solution (HBSS) then harvested from cell culture dishes using Hank’s-based cell dissociation buffer (Life Technologies) and further washed with PBS (pH 7.4, containing 5% (vol/vol) FBS) by centrifugation. The pelleted cells were resuspended by gentle vortexing in 70% cold ethanol (added drop-wise) and kept overnight at -20°C. The fixed cell suspensions were rinsed with cold PBS and permeabilized with 0.1% (wt/vol) saponin/ 5% (vol/vol) FBS/ PBS (pH 7.4) for 30 min, and stained with an anti-Ki67 antibody (dilution 1/100) for 1 h at 4°C. An Alexa Fluor 488-conjugated goat anti-rabbit IgG antibody was used as a secondary antibody (dilution 1/300). Ki67-positive cells were analysed by flow cytometry using FACSCanto II/ FACSDiva-Software (BD Biosciences).

### Confocal microscopy (immunocytochemistry of B2R)

Cells were grown on coverslips and fixed with 4% paraformaldehyde for 30 min at room temperature before proceeding to immunocytochemistry under permeabilized and non-permeabilized conditions (see FACS staining protocols described above). Non specific binding was blocked with PBS containing 5% goat serum and 5% fetal calf serum for 45 min at room temperature. Cells were then incubated with AS276-83 (2.5 µg/ml) followed by an incubation with a goat anti-rabbit fluorescein isothiocyanate (FITC)-conjugated IgG (2 µg/ml) (Santa Cruz Biotechnology). To visualize nuclei, the sections were counterstained with Hoechst 33258 (Molecular Probes). Coverslips were then mounted onto glass slides using FluoroGuard TM antifade reagent (Bio-Rad) and analyzed with a Multi Probe 2001 confocal argon laser-scanning system (Molecular Dynamics). Samples were imaged using a 60× differential interference contrast oil immersion objective lens on a Nikon TE300 microscope with a Bio-Rad Radiance 2000 confocal accessory. Images were collected by using the same Z values and were merged using a Silicon graphic software. Control experiments omitting the primary antibody displayed no staining (data not shown).

### Cellular permeability of B2RAs

Permeability of MDA-MB-231 cells to B2RAs was evaluated by either confocal microscopy or LC-MS/MS analyses. *Confocal* microscopy. MDA-MB-231 cells were grown to 70% confluence on round coverslips in 6 well plates containing medium (10% FBS/DMEM). Cells were then exposed to FITC-HOE 140 (10 µM) or FITC-dTat-HOE (10 µM) for 0, 15 and 60 min at 37°C in DMEM without FBS. At the end of treatments, cells were washed three times with PBS and then fixed with 4% paraformaldehyde for 30 min at room temperature. The presence and localization of the fluorescent staining was analyzed by confocal microscopy (FV1000, Olympus) coupled to an inverted microscope with a 63× oil immersion objective (Olympus). Mass spectrometry*.* Cells at 80% confluence in serum-free DMEM media were treated with a non-toxic concentration (2.5 µM) of antagonists at 37°C for the indicated times. At the end of treatments, cells underwent an acid wash to facilitate the release of surface bound ligands. Cell contents were released from hypotonic lysis using a 40:60 mixture of 15 mM Tris-HCl pH 7.4 / methanol. Cell lysates were next sonicated and subjected to protein precipitation following addition of 0.1% v/v formic acid. Precipitated proteins were removed by centrifugation and supernatants were concentrated using a Speedvac centrifugal evaporator. The sample was subjected to quantification using LC-MS/MS on a triple quadrupole mass spectrometer (Agilent G6410B). A calibration curve made from nine different concentrations of the reference compounds dissolved in suitable HPLC mobile phase solvents, was used to estimate antagonist intracellular content in each sample. The limit of quantification was 75 fmol (1 nM/75 µl).

### Drug-drug synergy experiments

Interactions (synergy, additive, antagonistic activities) between CP-B2RAs (FR173657 and NG68) and chemotherapeutic drugs (doxorubicin and paclitaxel) were evaluated using MTT standard assays. Twenty-four hours after seeding, cells were treated with CP-B2RAs, the other testing drug, or in combination. For combination testing, CP-B2RAs or the other testing drugs were added to plate in triplicate wells in ratios of IC_25_ (IC_25_A: IC_25_B), and cells are incubated in drug-treated medium for 72h and cell viability determined by MTT. Synergy was determined by calculating combination index (CI) value with the formula CI = (C_A,X_ / IC_x,A_) + (C_B,X_ / IC_x,B_), where C_A,X_ and C_B,X_ are concentrations of drug A and drug B used in combination to achieve x% drug effect. IC_x,A_ and IC_x,B_ are concentrations for single agents to achieve the same effect. A CI of less than, equal to, and more than 1 indicates synergy, additivity, and antagonism, respectively.

### Statistics

Data are presented as mean ± SEM. Statistical analyses were performed using either an unpaired Student’s *t*-test or a one-way ANOVA with a Dunnett post-hoc test, where appropriate. *p* values of < 0.05 were considered as significant.

## SUPPLEMENTARY MATERIALS FIGURES AND TABLE


