# Supplementary material for: Targeting intracellular B2 receptors using novel cell-penetrating antagonists to arrest growth and induce apoptosis in human triple-negative breast cancer
Source: Oncotarget. 2018 Jan 5;9(11):9885–906. doi: 10.18632/oncotarget.24009 (PMC5839409; doi:10.18632/oncotarget.24009)
Supplement: Supplementary file 1 [file oncotarget-09-9885-s001.pdf]

Targeting intracellular B2 receptors using novel cell-penetrating antagonists to arrest growth and induce apoptosis in human triple-negative breast cancer

SUPPLEMENTARY MATERIALS

cDNA SuperArray analysis

The GEArray Q-series array of Human Signal Transduction Pathway Finder (catalog OHS-014; SuperArray Bioscience) was used to quantify the expression of 113 genes representative of 18 signal transduction pathways. Quasi-confluent MDA-MB-231 cells were incubated or not with FR173657 (10 μM) in DMEM for 3 hours at 37°C. At the end of treatments, cells were collected, lysed and total RNA was purified using an ArrayGrade Total RNA Isolation kit according to the manufacturer's instructions (SuperArray Bioscience). For cDNA microarray analysis, equal amounts of cRNA (3 μg) were biotinylated, then the probes were hybridized onto the membranes containing specific genes for several transduction pathways. After extensive washing, specific hybridization onto the membranes was detected by chemiluminescence and revealed by autoradiography. A fold change cutoff of ≥1.5 as compared to control cells was set to identify regulated genes.

Supplementary Table 1: Marked gene expression changes in FR173657-treated MDA-MB-231 cells relative to untreated controls

Description	GenBank acc. number	Symbol	Fold change
**FR 173657 10 μM / 3h**			
NLR family, apoptosis inhibitory protein	NM_004536	NAIP	1,50
Bone morphogenetic protein 4	NM_130851	BMP4	1,50
Chemokine (C-C motif) ligand 20	NM_004591	CCL20	1,50
Cyclin-dependent kinase inhibitor 1B (p27, Kip1)	NM_004064	CDKN1B	1,70
Cyclin-dependent kinase inhibitor 2B (p15, inhibits CDK4)	NM_004936	CDKN2B	1,80
Cyclin-dependent kinase inhibitor 2C (p18, inhibits CDK4)	NM_078626	CDKN2C	2,20
Caudal type homeobox transcription factor 1	NM_001804	CDX1	1,80
CCAAT/enhancer binding protein (C/EBP), beta	NM_005194	CEBPB	1,70
Cathepsin D	NM_001909	CTSD	1,60
Chemokine (C-X-C motif) ligand 9	NM_002416	CXCL9	1,70
Early growth response 1	NM_001964	EGR1	1,60
Homeobox B1	NM_002144	HOXB1	1,60
Matrix metallopeptidase 10 (stromelysin 2)	NM_002425	MMP10	3,50
Platelet/endothelial cell adhesion molecule (CD31 antigen)	NM_000442	PECAM1	3,90
Nitric oxide synthase 2A (inducible, hepatocytes)	NM_000625	NOS2A	–1,50
Transferrin receptor (p90, CD71)	NM_003234	TFRC	–1,50

Note. Among the 113 genes contain in the transduction pathway microarray screen, 16 genes were found to be regulated (14 upregulated and 2 repressed targets) in response to a 3h-treatment with FR173657 (10 μM). Overall, the data generated from the transcriptional profiling study are, in general, in good agreement with the strong anticancer activity of FR173657 observed in MDA-MB-231 cells.

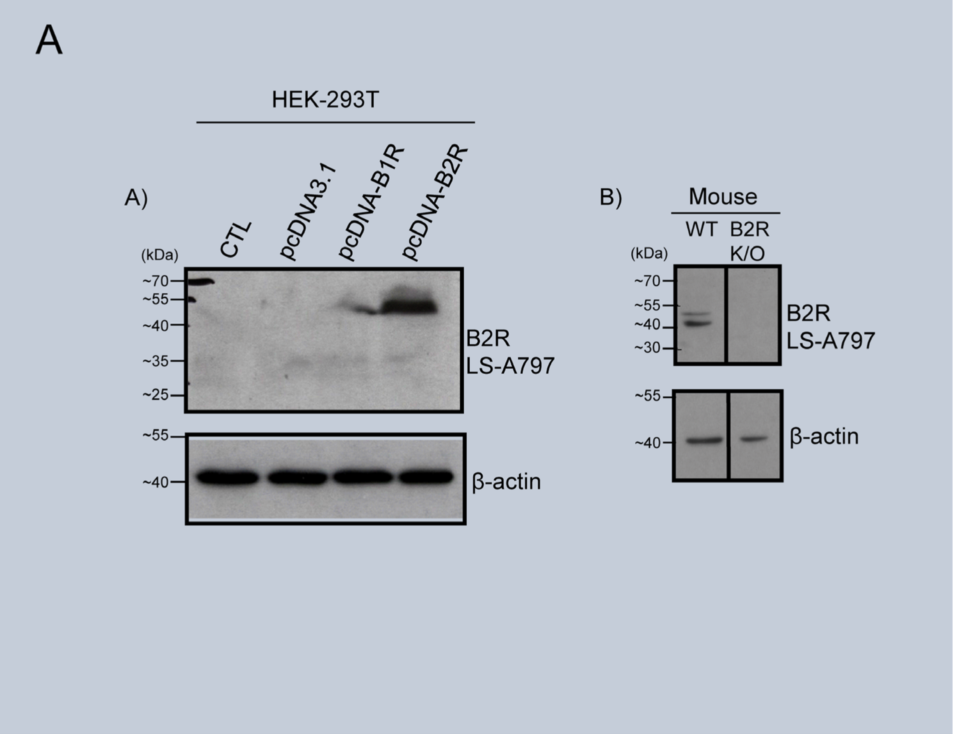
A
HEK-293T
A)
CTL
pcDNA3.1
pcDNA-B1R
pcDNA-B2R
(kDa)
~70
~55
~40
~35
~25
B2R
LS-A797
~55
~40
β-actin
B)
Mouse
WT
B2R
K/O
(kDa)
~70
~55
~40
~30
B2R
LS-A797
~55
~40
β-actin

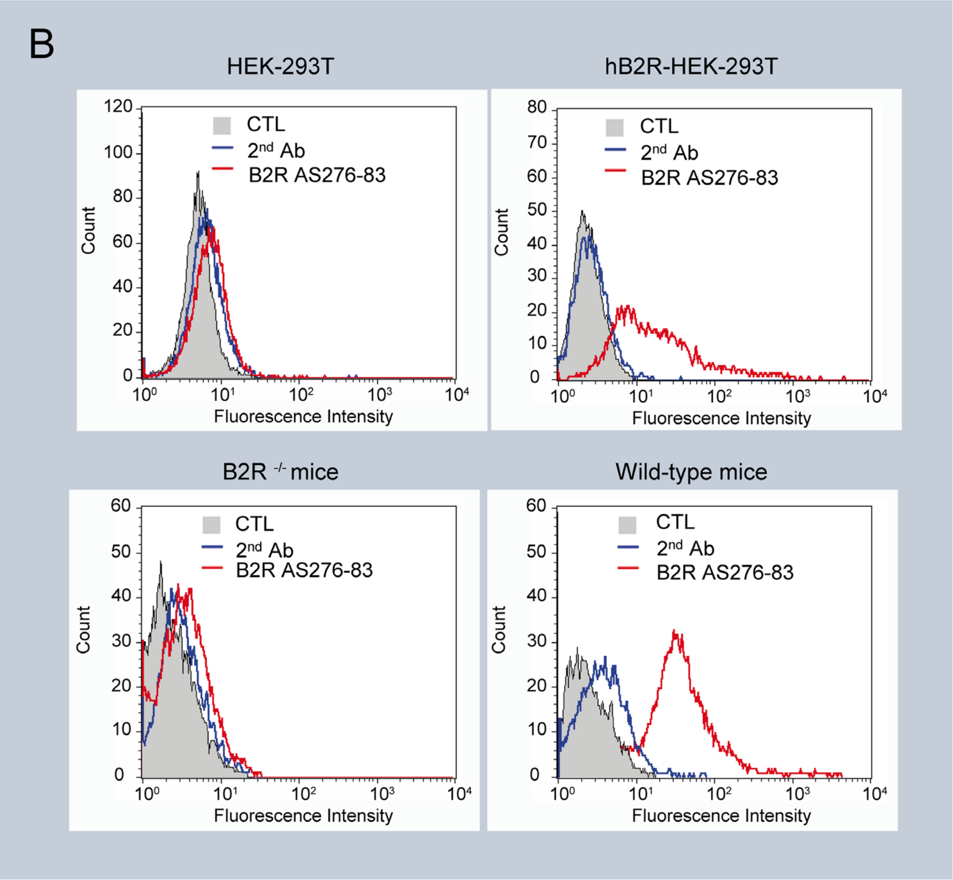
B
HEK-293T
hB2R-HEK-293T
B2R -/- mice
Wild-type mice
CTL
2nd Ab
B2R AS276-83
Count
Fluorescence Intensity

Supplementary Figure 1: Validation of target specificity of the two anti-B2R antibodies, LS-A797 (A) and AS276-83 (B), used in our study using transfected HEK-293T cells or in isolated hepatocytes of wild-type C57/Bl6 and B2R-KO mice, assessed by western blot and FACS analysis. (**A**) Western blotting demonstrating the specificity of LS-A797. Left panel: Cultured HEK-293T cells were transfected or not (CTL) with either pcDNA3.1 (vector), the pcDNA-B1R or the pcDNA-B2R. Protein cell extracts were prepared 48 h later. Right panel: Freshly isolated mouse liver hepatocytes were prepared with collagenase, as we previously described in refs [33, 77]. Equal amounts of denaturated protein extracts (10-50 µg) of HEK-293T cells and mouse hepatocytes were resolved by SDS-PAGE (9–12%) gel and transferred onto PVDF membranes, which were subsequently probed with the anti-B2R antibody LS-A797 (1:1000) and the β-actin antibody (1:10 000). Note that only the pcDNA-B2R cells (fourth lane) show a robust single immunoreactive band around 50 kDa. The occurrence of a partial immunoreactive band in lane three is due to a spill over of proteins from lane four into the adjacent well. (Right panel) B2R immunolabeling of hepatocytes with the same antibody was observed in wild-type but not B2R-KO mice. Representative autoradiograms of two to three independent experiments. (**B**) Binding specificities of AS276-83 towards cell-surface exposed B2R carried out by FACS using HEK-293T cells transfected or not with B2R (upper left and right panels) and freshly isolated hepatocytes from wild type and B2R-KO mice (bottom left and right panels). 5–10 × 10^6 cells per sample were suspended in PBS in the presence or absence of the mix antiserum AS276-83 (1:5000) at 4°C for 1h. Staining with omission of the primary antibody served as the negative control. Cells were washed then incubated with an anti-rabbit-Alexa488 (1:1000) at 4°C for an additional 1 h. Cells were then pelleted by centrifugation and resuspended in PBS containing propidium iodine (2.5 µg/ml) prior to FACS analysis (10^5 events/sample). The data shown are representative of two independent experiments.

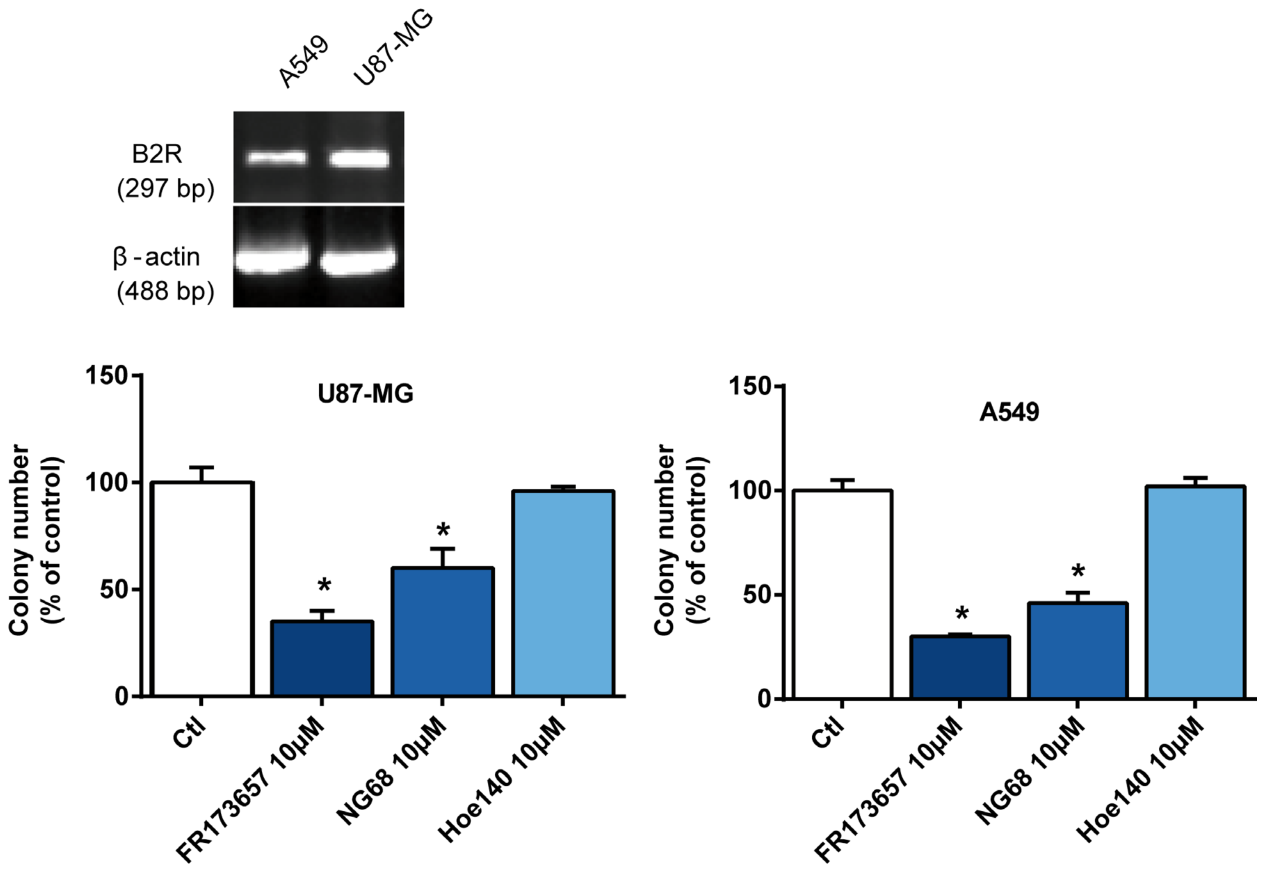

Supplementary Figure 2: Inhibition of clonal efficiency by permeant antagonist in B2R-expressing cancer cells. Upper panels: Representative ethidium bromide-stained 1% agarose gel showing PCR products. The RNA isolation, design of primers, and the PCR amplification protocols are described in ref. [34]. β-actin is shown as a loading control. Negative controls without RT showed no bands (data not shown). Bottom panels: Anchorage-dependent clonogenic assays with the B2RAs in the human cancer cell lines U87-MG (glioblastoma) and A549 (lung carcinoma) were performed as described in Figure 4 legend and under Materials. Data represent means ± s.e.m. of 4-6 experiments. $^{*}p < 0.05$ vs Ctl (Dunnett's test).

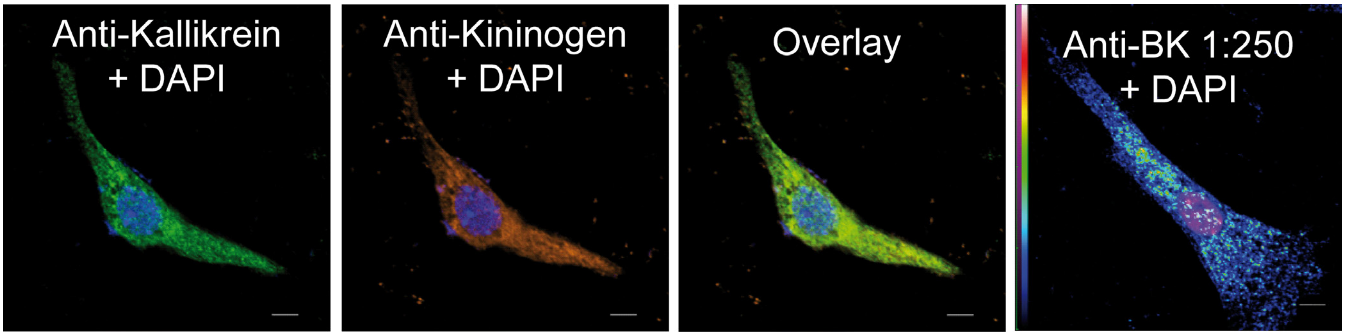

Supplementary Figure 3: Detection of tissue kallikreins, kininogens and BK-like immunoreactivities in MDA-MB-231 cells assessed by confocal microscopy. Immunostaining and confocal microscopy were carried out using similar experimental design as described in Materials and Methods and in legends to the other figures. In brief, cells cultured on glass coverslips were fixed (2% PF, 10 min at RT), permeabilized and blocked (PBS pH 7.4/ 0.1 % saponin/ 5% goat serum, 30 min at RT) and then incubated 1h at RT with the primary antibodies at the following dilutions: mouse monoclonal antibody anti-human kininogens (1:200), rabbit polyclonal anti-tissue kallikreins (1:200) and anti-BK (1:200) antisera. Nuclei were counterstained with DAPI. Left and middle panels: Representative confocal midsection micrographs showing the presence and co-localisation of endogenous kallikreins (green) and kininogens (red) not only in the cytoplasmic region, but also in the intranuclear region (dual signal overlay). Also presented, are confocal images of MDA-MB-231 cells showing positive BK immunoreactivity (right panel), characterized by a fine granular speckled cytoplasmic and nuclear staining. Pseudocolor fluorescence intensity scale as in Figure 3. No fluorescent signals were detected in the absence of primary antibody (data not shown). Note the anti-human kininogen antibody used in our study reacts with a common epitope present both on the high and low molecular weight kininogens (i.e. domain 1), it is therefore not possible to ascribe the fluorescent signals observed with this antibody to a particular kininogen species. We also cannot rule out that anti-BK antibody displays some cross-reactivity with the precursor form kininogen containing the epitope recognition. Scale bar = 5 µm.

HOE 140 ($C_{59}H_{89}N_{19}O_{13}S_1$)
MS calculated: 1 304.52
MS(ESI) found: 1 305.8 (M+1), 653.7 (M+2)/2, 436.4 (M+3)/3

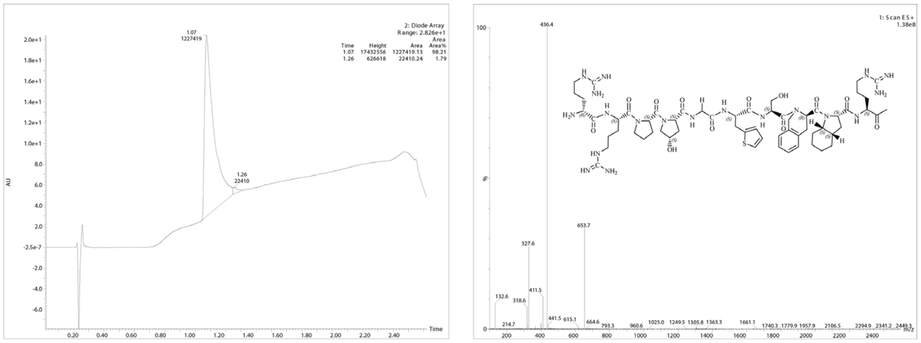

NG68 ($C_{120}H_{205}N_{53}O_{27}S_1$)
MS calculated: 2 854.31
MS(ESI) found: 952.9 (M+3)/3, 715.2 (M+4)/4, 572.5 (M+5)/5, 477.3 (M+6)/6, 409.5 (M+7)/7

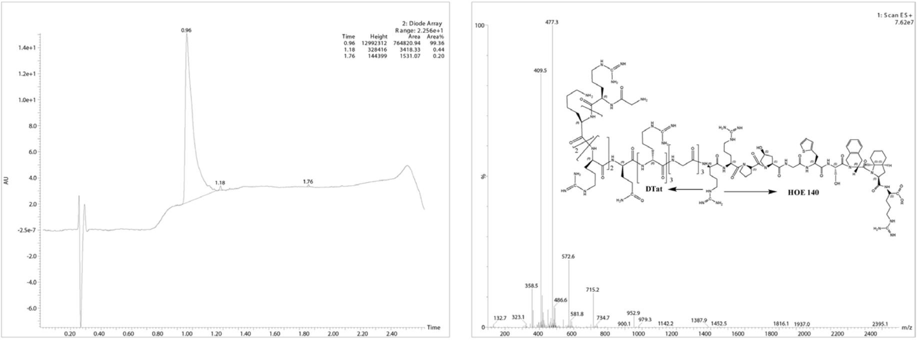

NG134 ($C_{83}H_{127}N_{19}O_{17}S_1$)
MS calculated: 1 695.07
MS(ESI) found: 1 696.5 (M+1), 849.0 (M+2)/2, 566.6 (M+3)/3

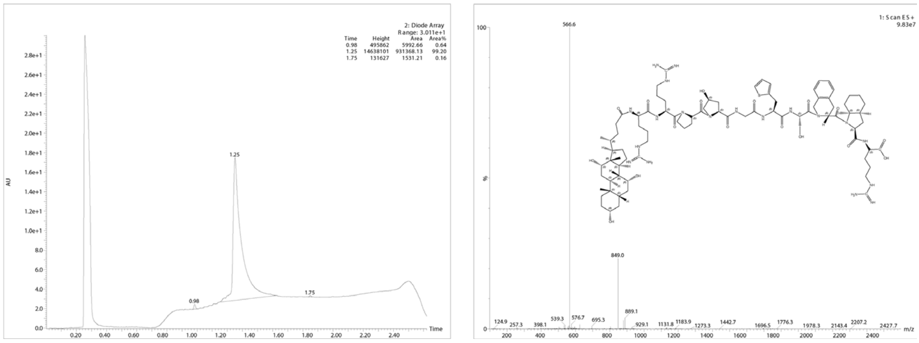

Supplementary Figure 4: HPLC and Mass spectrometry analyses of B2RA peptides used in the study. Peptides were purified by reverse phase HPLC on Waters 2535 module (with Waters 2489 UV detector) with a preparative ACE C18 (5 µm, 250 x 21.2 mm) column. The pure product fractions were combined and lyophilized. Compound identification and purity was assessed by analytical UPLC-MS (Waters Aquity H-Class, SQD2 (ESI) mass detector) with a Waters BEH C18 (1.7 µm, 2.1 x 50 mm) column. According to UPLC-MS analysis, the purity of the peptides exceeded 98%.
